# Consolidating a framework of autistic camouflaging strategies: An integrative systematic review

**DOI:** 10.1177/13623613251335472

**Published:** 2025-05-30

**Authors:** Jacques Nel, Maxine Spedding, Susan Malcolm-Smith

**Affiliations:** University of Cape Town, South Africa

**Keywords:** autism spectrum, camouflaging, framework, masking, systematic review, thematic meta-synthesis

## Abstract

Autistic ‘social camouflaging’ research is proliferating. However, the term is multi-definitional. Our integrative systematic review (CRD42022324957) pursued a single-framework, qualitative meta-synthesis of camouflaging in autistic adults. We report specifically on different types of camouflaging strategies and the contextual factors that moderate them. A total of 2346 articles were extracted from online databases, ‘Connected Papers’ and citation searching – reflecting qualitative data from diagnostically confirmed, or self-identifying, autistic individuals. The results of 28 studies were coded and thematically synthesised, and included 2669 men, women and non-binary individuals above the age of 16. We noted six types of strategies and four contextual factors that modulate them. ‘Imitation’ played a notable role in facilitating further learning and development of camouflaging strategies, thus shifting one from behavioural suppression to sophisticated compensations. The results encourage researchers to place camouflaging data within this (or other) frameworks. Limitations include data reflecting only a subset of the autistic community, skewed towards women and the United Kingdom, raising questions about the impact that gender and UK culture has on our current understanding of camouflaging.

**Lay abstract:**

Research into autistic ‘social camouflaging’ is gaining momentum. However, with so many different definitions, the term needs better clarification. We thus reviewed existing articles that discuss camouflaging, in order to bring all of the different understandings of adult autistic camouflaging together into a single framework. This article focuses on findings related to different types of camouflaging *strategies*, and the types of situations that help or hinder individuals when they use such strategies. After screening 2346 articles (which were listed on online research databases) – we arrived at 28 articles that were included in this study. Data were based on the personal reports of 2669 adults (over the age of 16 years) with confirmed, or self-identified, autism. These reports were in English, qualitative, published papers. We noted six types of camouflaging strategies, and four situational factors that influence them. ‘Imitation’ was noted as a key means by which strategies develop. The results encourage researchers to build on frameworks such as ours. We also found that much of the sample consisted of women from the United Kingdom, and thus, there is a question of how much influence UK culture has on our current picture of camouflaging.

Autism is a neurodevelopmental status which reflects diverse social needs, sensory processing and systemising habits (e.g. a need for structure and predictability) – when compared to societal majority norms (American Psychological Association (APA), 2022; Baron-Cohen, 2009). Autistic individuals are thus a neurominority population, as their needs may place them at odds with dominant (neurotypical) social patterns. While autism is a categorical status (i.e. a shared profile meeting all criteria), some people may be sub-clinical. Thus, they do not present *outwardly* the degree of difficulty that warrants formal diagnosis, and so may go unnoticed (Chown & Leatherland, 2020; Pearson & Rose, 2021). Yet, their inner experiences and challenges may be similar. Thus, we use ‘autism’ instead of Autism Spectrum Disorder (ASD; APA, 2022).

Autistic camouflaging means that one obscures their autism-linked social difficulties and neuro-minority status, with the goal of social integration and avoiding adversity (e.g. Libsack et al., 2021; Pearson & Rose, 2021). Autistic individuals experience exclusion, discrimination, infantilisation and abuse (Botha et al., 2020; Hull et al., 2017; Livingston, Shah, & Happé, 2019), due to society’s negative perceptions of difference (Pearson & Rose, 2021). Hiding difference is thus self-protective against the above (e.g. Libsack et al., 2021). This includes suppressing organic responses like stims, performing ‘typical’ behaviours like eye contact and using cognitive heuristics (Hull et al., 2020; Livingston, Shah, & Happé, 2019; Pearson & Rose, 2021). The *Diagnostic and Statistical Manual of Mental Disorders* (5th ed., text rev.; DSM-5-TR) likewise notes that learned strategies may mask autistic symptoms (APA, 2022, p. 58).

The impact of camouflaging can be concerning. There is a well-established high rate of depression, burnout and suicidality among autistic individuals (e.g. Cassidy et al., 2022; Cook, Hull, et al., 2021; Hull et al., 2020; Libsack et al., 2021). This may be exacerbated by fallout from camouflaging itself (Cook, Hull, et al., 2021; Hull et al., 2020; Pearson & Rose, 2021). While such links are inconsistently reported, a meta-analysis by Khudiakova et al. (2024) found a significant moderate correlation between camouflaging and negative mental health outcomes. Possible attributions included exhaustion and anxiety from constant behavioural monitoring, related autistic burnout as a consequence of navigating neurotypical spaces, the strain attached to specific forms of camouflaging or a sense of inauthenticity that has a deleterious effect on self-esteem. Yet, camouflaging severity may merely be a marker of the level of stigma and discrimination an individual experiences, which itself could directly account for poorer mental health outcomes (Khudiakova et al., 2024). All the while, difficulties remain undetected, without support.

There is thus a clear need to conceptually clarify camouflaging, as it is a multi-dimensional construct, with varied operationalisations (e.g. internal intent to camouflage vs externally evident attempts) (e.g. Fombonne, 2020; Lai et al., 2021; Libsack et al., 2021; Williams, 2022). Bolstering of screening, and awareness of experiential facets of camouflaging, would enable clinicians to improve support of autistic patients. Of the existing autism reviews to date, several focus on female-specific camouflaging (Allely, 2019; Hull et al., 2020; Tubío-Fungueiriño et al., 2021), on *how* camouflaging is researched and reported on (Cook, Hull, et al., 2021; Hull et al., 2020; Libsack et al., 2021), on the sex-mediated differences between autistic men and women (Lai et al., 2015) and on the link between camouflaging experiences, motives and mental health outcomes (Field et al., 2024; Zhuang et al., 2023). Many have outlined different camouflaging categories. Yet, to our knowledge, this review is the first to systematically consolidate and delineate all strategy types.

We interpreted results through a Bayesian lens of social cognition. Bayesian learning describes how we experiment with the environment and gather data, which is iteratively used to build predictions of the world (e.g. Amoruso et al., 2019). For example, a child may socially learn by trying new behaviours or testing new boundaries and seeing the consequences. Such data develops into social templates that guide behaviour in a specific situation or context (Ai et al., 2022). One facet of social cognition built in this way is Theory of Mind (ToM) (Gopnik & Wellman, 2012), or learning to consciously predict others’ mental states. This may reflect the most complex outcome of Bayesian learning in camouflaging.

To be clear, autism does not by definition reflect defective ToM (Gernsbacher & Yergeau, 2019). What is rather suggested is that when a neurotypical and neurodiverse person interact, both must work to understand the other through inference. This is comparable to a cultural divide – but owing to the social power of the neurotypical individual, the neurodiverse individual is forced to bridge the entire gap (Ai et al., 2022). Some may navigate such interactions fluidly, some may experience such efforts as deliberate and taxing (e.g. Gould, 2017) and some may only be able to engage in a ‘superficial’ performance of neurotypicality, with issues in generalising templates (Hull et al., 2020; Senju, 2012). As shown below, camouflaging reflects a repertoire of many kinds of strategies, with varying complexities. The benefit of a Bayesian lens is that it describes how social data is stored and consciously used in a *top-down* manner in increasingly complex, contextualised ways.

We aimed to synthesise qualitative conceptions of camouflaging among autistic adults into a single framework and to discern the breadth and agreement of camouflaging strategy types across the literature. In this article, we report on camouflaging (1) strategy types and (2) related contextual factors, within a social cognitive framework. While a third domain (motivations) was investigated in our broader study, the results are not reported here. Our questions were:

• What are the types of strategies seen in adult autistic camouflaging?
• What are the contextual moderators of strategy effectiveness in autistic adults?

## Method

Following Preferred Reporting Items for Systematic Reviews and Meta-Analyses (PRISMA; 2022) guidelines, we conducted an ‘integrative’/critical literature review (De Klerk & Pretorius, 2019; Grant & Booth, 2009). This is a form of systematic review that goes beyond narrative summation, towards the meta-synthesis or meta-analysis of data, and thus the creation of unified frameworks (Torraco, 2005). We opted for the meta-synthesis of qualitative data in the form of an adapted thematic analysis (De Klerk & Pretorius, 2019) with data coded into themes (Torraco, 2005; Whittemore & Knafl, 2005).

### Eligibility

We used the **S.P-I.D.E.R**. inclusion framework (Sample, Phenomenon of Interest, Design, Experience, Research-type) (University of London, 2022):

**(S.)** Adults (16+ years) with confirmed or self-identified autism.
**(P-I.)** Any discussion of camouflaging (appearing less autistic/socially different).
**(D.)** Empirical studies (i.e. novel data generation or integration).
**(E.)** Outcome variables were: (a) camouflaging strategy types, (b) motivations and (c) contextual factors. Articles had to address any one(+) domain (a–c), and at least three specific examples to ensure topic-focus.
**(R.)** Any form of qualitative data (including text analyses or semi-qualitative surveys) that came from full-text, peer-reviewed English articles. We excluded articles with poor reporting quality (see Quality Assessment).

### Data sourcing

**Figure 1.**
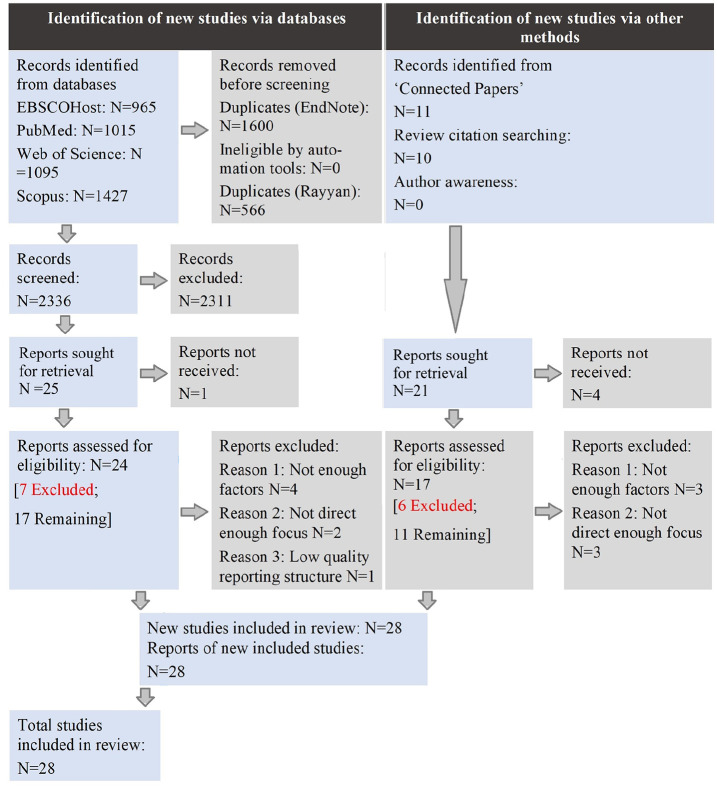
Article selection process, reported per PRISMA (2022) standards.

We utilised (a) database searching (EBSCOhost (Academic Search Premier, CINAHL, APA PsychInfo, APA PsychArticles), PubMed, Web of Science and Scopus (including Embase)), (b) the ‘Connected Papers’ (2022) platform and (c) citation searching reviews/editorials. A combination of free-text and controlled terms was used (Evidence for Policy and Practice Information and Co-Ordinating Centre, 2010) (see Supplementaries). Database articles (26 April 2022) were exported to Rayyan.AI (Ouzzani et al., 2016) for blind co-screening. This occurred in stages, by title, abstract and full-text levels (De Klerk & Pretorius, 2019). The first author screened all articles. The second and third authors screened 510 articles without overlap (>20%; Schlosser et al., 2007), at title and abstract levels. This achieved an acceptable Cohen’s kappa of 0.77 (McHugh, 2012), and through discussion, we reached full consensus. ‘Connected Papers’ was searched 17–19 May 2022 using qualitative articles centred on camouflaging (i.e. Beck et al., 2020; Bernadin et al., 2021; Cage & Troxell-Whitman, 2019; Cook, Crane, et al., 2021; Cook et al., 2018, 2022; Hull et al., 2017; Livingston, Shah, & Happé, 2019; Miller et al., 2021; Schneid & Raz, 2020). The article selection process described above is represented in Figure 1.

### Quality assessment

While using peer-reviewed articles assisted in quality assurance, we further assessed reporting quality using the Lorenc-adjusted Hawker et al. (2002, as cited in Lorenc et al., 2014; see Supplementaries) framework. We applied a weighting approach towards a best-evidence synthesis (Schlosser et al., 2007). High-quality (**A-Graded**) studies were used to derive core themes, with the remainder (**B-Graded**) being supplemental. **C-Graded** studies were considered too low for inclusion. Articles were ranked individually and then cross-compared per item to ensure fair comparative scoring.

### Data analysis

Data were extracted into a pre-designed spreadsheet. The second and third authors reviewed the initial codes and extractable factors (including sample size, age, gender, nationality, study-, method-, respondent- and diagnostic-type) in a group discussion. Thomas and Harden (2008), and Lachal et al. (2017) suggest a qualitative meta-synthesis that line-codes article ‘results sections’ as transcripts. Thus, we conducted a semi-inductive thematic analysis (De Klerk & Pretorius, 2019) in QDA Miner Lite using the Braun and Clarke framework (Terry et al., 2017). The study was semi-inductive in that certain established concepts were drawn on when creating a provisional set of codes (e.g. imitation, scripting, hiding stimming) – following initial familiarisation. However, these codes were revised, expanded or nested iteratively so as to provide flexibility. Codes were clustered into group-reviewed themes and hierarchies and then cross-checked with each original article to ensure no significant contradictions. Themes were then interpreted per a social cognitive (Bayesian) framework.

### Quality assurance

To ensure **clarity and auditability** (Booth et al., 2012), all steps and rationales are outlined. Librarian consultation helped reduce **database bias**; however, the results are **publication biased** (Schlosser et al., 2007). **Selection bias** was countered by co-scoring and supervisory discussions (Schlosser et al., 2007). For confidence in the **certainty** of results, we scored *moderate* confidence using GRADE-CERQual (2023), due to high agreement in the dataset, but a demographic bias that impacts generalisability. The study received departmental clearance from the University of Cape Town and is listed on Prospero (CRD42022324957).

## Results

We included 28 studies, with a total of 2450 participants contributing to the analysis. We narrowed the sample to focus on participants who provided qualitative data. However, two articles only reported demographics for their entire sample, not specifically the subset contributing to the qualitative data. As a result, the final demographic data had to be based on a maximum of 2669 participants to account for this limitation.

All demographic factors were reported inconsistently across studies. Thus, we provide the *total* and *reported total* of a given factor. *Totals* are based on the maximum 2699 participants, while *reported totals* are based on only the number of participants for which a factor was reported. For example, if X people identified as male, but gender was only reported for 2000 participants, this reflects a *total* of X/2699, and a *reported total* of X/2000.

Data were analysed for camouflaging strategies and their contextual moderators. We reference articles per their number# in Table 1. For detailed examples, see Supplementaries. While a given theme may not be *unique* to autism, the results integrate the existing autism-based literature.

**Table 1.** Included articles for analysis.

	Authors	Design	Diagnostic status
	Quality: A-Graded		
1	Bargiela et al. (2016)	Survey and Single Interview	Formal Diagnosis
2	Bernadin et al. (2021)	Survey and Single Interview	Formal Diagnosis
3	Botha et al. (2020)	Single Interview	Formal Diagnosis and Self-identified
4	Bradley et al. (2021)	Survey	Formal Diagnosis and Self-identified
5	Cage et al. (2018)	Survey	Formal Diagnosis and Self-identified
6	Cage and Troxell-Whitman (2019)	Survey	Formal Diagnosis
7	Collis et al. (2022)	Survey and Single Interview	Formal Diagnosis
8	Cook et al. (2018)	Single Interview (triangulated dyads)	Formal Diagnosis
9	Cook, Crane, et al. (2021)	Single Interview	Formal Diagnosis
10	Crompton et al. (2020)	Single Interview	Formal Diagnosis
11	Harmens et al. (2022)	Text Analysis [blogs]	Formal Diagnosis and Self-identified
12	Hickey et al. (2018)	Single Interview	Formal Diagnosis
13	Hull et al. (2017)	Survey	Formal Diagnosis
14	Kapp et al. (2019)	Single Interview and Group Interview	Formal Diagnosis
15	Leedham et al. (2020)	Survey	Formal Diagnosis
16	Lilley et al. (2021)	Single Interview	Formal Diagnosis
17	Livingston, Shah, and Happé (2019)	Survey	Formal Diagnosis and Self-identified
18	Mantzalas et al. (2022)	Text Analysis [social media]	Unclear
19	Miller et al. (2021)	Survey	Unclear
20	Milton and Sims (2016)	Text Analysis [magazines]	Unclear
21	Schneid and Raz (2020)	Single Interview	Formal Diagnosis and Self-identified
22	Tint and Weiss (2018)	Group Interview	Formal Diagnosis
	Quality: B-Graded		
23	Baldwin and Costley (2015)	Survey	Formal Diagnosis
24	Dachez and Ndobo (2018)	Single Interview	Formal Diagnosis
25	Davidson and Henderson (2010)	Text Analysis	Unclear
26	Forster and Pearson (2020)	Single Interview	Formal Diagnosis
27	Howard and Sedgewick (2021)	Survey	Formal Diagnosis and Self-identified
28	Milner et al. (2019)	Single Interview and Group Interview	Formal Diagnosis and Self-identified

### Research question 1: camouflaging strategy-types

See Table 3 for behavioural examples of each type of strategy.

#### Theme 1: masking

The mask reflected (a) something behind which a ‘true self’^12^ is intentionally hidden^16^ through suppression, while (b) something was displayed outwardly to the world,^13^ like a persona. It thus encompassed hiding signs of difference and being other than oneself when socialising.^13^ Often as a response to stigma and shaming, this helped avoid pathologisation or abuse from others.^3,6,17,20,25^

##### Subtheme 1.1: suppression

**Suppression**^9,13^ reflected becoming still or holding-back,^3^ minimising one’s speech^13^ or ‘reduc[ing onself . . .] to the point of suppressing [natural] reactions’.^21^ In potentially threatening situations, such strategies helped to avoid standing out.^17,18^ As one individual put it: ‘I remain silent when I might otherwise have spoken, knowing that I can’t always tell whether or not my comments would be welcome’.^13^ More moderate versions of this included self-editing by suppressing aspects of oneself,^9,19^ versus almost entirely eliminating noteworthy acts altogether. A facet herein was ***1.1.1. hiding personal information, needs, and preferences***, which involved hiding one’s personhood. Individuals felt forced to minimise their preferences,^10,16^ which might be treated as odd or inconvenient, including not speaking of personal (potentially unconventional) interests.^9,13^ They also withhold personal information which might be poorly received.^13^

Another facet was ***1.1.2. hiding distress***, for fear that others would invalidate^15^ or gaslight^11^ one’s distress as overreactions. People masked difficulties^13,22^ and worked ‘hard to hold [themselves] together’,^11^ while internally ‘driving [themselves] crazy’^22^ (e.g. being composed at school, but with frequent ‘meltdowns’ at home^1^). Individuals also masked ‘sensory differences such as pain due to sounds’,^19^ or distress at the actions of others.^7^ One participant noted that at the local shopping centre ‘the noise and activity is extreme. I have a meltdown (carefully hidden as much as possible) every week on average’.^23^ Relatedly, we noted complete ***1.1.3. suppression of stims***,^14,19^ or reducing^7^/transmuting stims into acceptable,^14^ subtle^7,13,19^ forms. Self-monitoring and self-policing formed key mechanisms here.^7^

##### Subtheme 1. 2: performativity

**Performativity** reflected part- or whole-performance^12^ of personas^1,17^ that were identity-incongruent^15^ (e.g. feigning enjoyment of conversations^1,7^). It allowed for the gaps left by suppression to be filled by neurotypically expected behaviours. Personas were a ‘social mode’,^4^ ‘special personality’^21^ or – in theatre-terms^24^ – a *character*. Individuals played a role,^4,16^ game^4^ or part,^4,13^ and even treated their ‘clothes rather like a costume’^13^ or a ‘person that [they] put on’.^15^ Some described specific personas, such as ‘bubbly and vivacious’^1^ or ‘mister footloose’,^15^ while others reported being many personas.^11,16^

Sometimes, personas were ‘pretending to be someone else’ specific^1^ or a ‘borrowed personality’,^24^ linking to ‘Imitation’ (below). At the extreme, performing made individuals feel like they were stripped of a sense of identity,^1,11,17^ with their presentation being ‘something contrived’,^21^ a ‘patchwork of acts’^4^ or ‘bits and pieces of disconnected things’.^16^ They felt alienated from their ‘true’ self behind the mask. Distress was highlighted in terms of personas helping one ‘to accomplish much that is considered normal, successful, desirable. But this is a shell, and within it I’m bombarded and puzzled’.^25^ Likewise, it feels ‘like the weight of a black cloud is hanging on me having to be this fake version of me’.^13^ However, a more moderate version of a persona sometimes included being an edited version of oneself.^13^ Such variations might relate to situational roles, as people might edit behaviour according to the setting (e.g. the model pupil,^1^ a ‘professional mode’,^25^ ‘the mediator’^21^).

#### Theme 2: tools for a growing repertoire

This section outlines how autistic individuals may develop their strategies. Instead of just implicit tracking of social signals, socialising is a conscious skillset that is developed.^26^

##### Subtheme 2.1: imitation

**Imitation**, as a strategy, reflected (a) live mirroring^19^ of one’s social partners. One person noted intuitively and ‘automatically mimic[king] what other people are doing, what people are saying, [and] how people say things’.^1^ This created neurotypically passable behaviours. Yet, it could be dangerous if one unintentionally mirrored interactional undertones, such as ‘flirtatious behaviour’.^1^

However, (b) as a *tool* of observation and mimicry,^22^ one could either strategically copy those in front of them^13^ or observe the way others engaged with each other.^9^ They then developed a repertoire of behaviours picked up from others.^1^ It is important to spotlight here how the performance of imitative strategies itself, more than any other form of camouflaging, created a context for further, increasing, or more nuanced camouflaging. Individuals might delay emulation, and later trial^9^ and refine strategies^17^/repertoires through an iterative process.^9^ This linked with ‘Performativity’, as one could adopt the fashion or interests of others. Yet, such observational efforts may feel frustrating and alienating, and one person commented that neurotypical individuals ‘never had to study autistic people in the same way I study them’.^10^ This speaks to the ‘double empathy’ discourse (Milton, 2012), in which neurotypicals’ lack of understanding of autistic communication equally contributes to communicative breakdown.

Individuals might pick role-models (e.g. perceived socially successful/valued people^9,13^) in their proximity or through media. In ***2.1.1. learning from media***, repertoires were built through TV, movies^13^ and ‘reading different books’^16^ or magazines.^1^ Individuals might learn masking from these sources, seeing how characters had specific ways of dealing with specific, relatable situations, and drawing inspiration from them.^1^

##### Subtheme 2.2: active social training

Coaching boosted social strategies. This included self-study materials, such as ‘books on body language’ or ‘a guide to being assertive’.^1^ There was utility in contextualised social training (e.g. ‘especially around bullying’^20^), reflecting a benefit to learning to draw on such repertoires. While such coaching could be helpful in building strategies, the darker side reflected skills being *forced* on individuals or prescribed by those around them, ‘promoting rigid concealment’^25^ to a coercive and insulting^21^ degree.

#### Theme 3: cognitive strategies

These strategies are geared towards helping bridge social communication gaps^13^ when faced with neurotypical social standards. This was achieved by way of intellectually^17^ conceived rules,^4^ heuristics and templates. In tracking social patterns, individuals adopted guidelines or structured techniques to reduce situational uncertainty,^13^ as every social situation required decoding and interpretation.^25^ This included laughing at joke cues,^17^ over-emphasising interest,^13^ ‘nodding, agreeing’ or making ‘generic comments’.^13^ One specific rule was to ask questions of people^13^ about themselves,^17^ flipping conversation back with an: ‘And you?’.^9^

##### Subtheme 3.1: scripting

**Scripting** reflected social preparation (e.g. of niceties,^17^ phrases^9^ or ‘a list of public responses’^23^), with simple, typically more inflexible responses stored up ahead of time.^17^ Scripting might include ‘one or two questions’, conversational topics, anecdotes and potential responses.^13^ Individuals might imagine ‘how whole conversations might go’.^13^ Taken digitally, preparation emerged in written communication,^1^ as there was ‘time to think about what they want[ed] to say’,^27^ and to ‘research templates’ or borrow phrases.^27^

##### Subtheme 3.2: modulation

**Modulation** was behavioural adaptation^4^ that, while not organic, was not *necessarily* identity-incongruent (thus distinguished from Performativity, which included degrees of inauthenticity to one’s identity). This is thus the distinction between playing a role and choosing which side of oneself others are allowed to see. Individuals curated a particular image^6^ or desirable^17^ output – such as being good,^1^ nice,^9^ ‘more extroverted’^2^ or demonstrating their competence, skill^6^ and positive attributes.^9^ Thus, this subtheme arguably reflected more global (and neurotypically shared) impression management goals, and sharing a sense of similarity to one’s social partner,^9^ such as through highlighting normative interests.^9^ These efforts could be identity-congruent as, for example, if one’s usual facial expressions read as hostile, they might over-emphasise and ‘act the good will that [they] genuinely [felt]’^13^ to relay their internal state. Modulations might also include forcing (approximate) eye contact,^9,13,17,24,28^ using the ‘right facial expression’^4^ and ‘tone of voice’.^27^

##### Subtheme 3.3: mental equations

**Mental Equations** denoted live, fluid^17^ mentalising (ToM) activities to predictively^13^ track interactions. For those who consciously built up intricate mentalising frameworks, this reflected an unintuitive yet ‘intellectual mode’^24^ in which they drew on pattern detection and data templating^17^ to learn ‘how to read other people’.^26^ Input was used formulaically,^26^ as the social partner’s ‘gesture + facial expression + context’ was used to derive their mental state.^17^ By solving social ‘mathematical equations’,^4^ the resulting templates could be stored and refined.^24^ Templates might be bespoke,^4^ as ‘the more you get to know someone the easier it is to read them’.^26^ Mental equations, in turn, supported other strategies, such as ‘Scripting’ through knowing someone’s interests.^19^ Ultimately, Mental Equations signalled true social cognition. Yet not everyone developed such intricate rules.^13^ They also caused strain, with one individual reporting how ‘it’s very draining trying to figure out everything all the time[,. . .] where you have to type every command in’.^1^

The next set of supplementary strategies overlapped, and interplayed, with contextual moderators, as they would shift the nature or context of an interaction in some form.

#### Theme 4: use of others

Camouflaging included using others as confidantes, for supported communication,^27^ whereby they checked writing or provided live support.^27^ Neurotypical people might support by explaining what was unfolding in a situation,^10^ or ‘tak[ing] the lead in situations’ where the autistic individual was uncomfortable.^15^ This was echoed in the comment that ‘if it’s a really good friend she would explain [the situation] to me. It’s the difference of asking for help in a focused and fully aware manner, versus being lost without even knowing why you’re lost’.^21^ However, it did not always include the other’s awareness. Some described attaching to someone more social ‘to give the illusion of being sociable’^4^ or enlisting an unwitting other by pretending to want their company.^25^ Although Schneid and Raz (2020) viewed assistance as an alternative to camouflaging, it also neatly reflected a supplement to camouflaging in the dataset.

#### Theme 5: ‘unhealthy’ practices

People might adopt unhealthy practices to bolster their social image or as a social lubricant. For the former, one person’s attempts to fit in included restrictive eating and struggling with resultant anorexia nervosa.^19^ For the latter, alcohol functioned as a crutch to ‘free up’^1^ the individual to get through events and ‘feel less anxious and self-aware’.^4^ Thus, substances might bolster camouflaging, as without them ‘masking [might] become more difficult’.^19^ ‘Substance use’ also linked to ‘Performativity’, as one person explained how ‘although they’d not been drinking[,..they] let [the others] think that’^10^ to excuse possible awkwardness.

#### Theme 6: selective settings

This denotes *accommodation* (Livingston et al., 2020). Some pursued environments that supported strategy effectiveness,^17^ including through community norms such as the ‘reserve [*and*] reticence’ of the United Kingdom,^17^ slow and deliberate country-lifestyles,^20^ or ‘straightforward’ male interactions.^1^ Individuals found structured settings such as journal clubs^12^ where there were clearer, topical frameworks that guided interaction, or else environments that played to one’s skills, such as less social workplaces.^17^ Also, other people of a different age, socio-professional class or nationality^24^ were often different enough from the autistic person that, in befriending them, ‘there could be no [direct] social comparison’.^24^

### Research question 2: contextual moderators of camouflaging

Note that when investigating whether camouflaging was implicated in specific settings, the overall data suggested any given environment could elicit camouflaging.

#### Theme 1: unclear and unexpected cues

Given the inflexibility of some strategies, predictability supported sustained ‘passing’ in neurotypically dominated settings. Strategies might falter during confusing interaction,^15^ thus individuals might not ‘like interacting with people [they] can’t “read”’,^23^ implicating Cognitive Strategies. As one participant noted: ‘I am stuck when I meet people who have no interests and extreme extroverts’.^17^ Less complex cognitive strategies might not transfer well into novel situations^17^ (e.g. scripts not being useful to handle unexpected conversational pivots^17^). In unstructured settings like parties, cognitive strategies could also be too slow, straining and/or inflexible for the quick-paced interactions.^17^ This leads to the next point.

#### Theme 2: number of people

Socialising in large groups^1^ further contributed to such unstructured pressures, making it ‘difficult to talk to people’^12^ unless a confidante was available to serve as a social foil, and thus as one person put: ‘Social grouping brings out my autism’.^21^ This has been understood by the fact that groups were very demanding on cognitive resources due to the sheer number of social cues one had to track.^17^ Interestingly, ‘Performativity’ in terms of adopting a persona was helpful in such a setting.^1^ Furthermore, ‘Suppression of Stims’ occurred even in one single person’s presence, with individuals preferring to stim ‘out of sight’^7^ due to being made to feel belittled, awkward and reprimanded when doing so in front of neurotypical others.

#### Theme 3: distress and overwhelm

In high-demand settings,^17^ increased stress might lead to breakdowns. This included challenging sensory environments^17^ where, for example, auditory insults made masking tougher.^19^ Flare-ups in anxiety^22^ deteriorated one’s capacity to ‘cope’, putting individuals at risk of a meltdown,^4^ and thus they could not continue to mask the strain any longer.^22^ In a longitudinal sense, as life demands and stressors escalated into adulthood, strategies sometimes became insufficient^17^ due to exhaustion and burnout. This especially impacted on ‘Cognitive Strategies’ once again, as overwhelm meant that one ‘[does not] have the energy to really try (to interpret meanings)’.^26^ Distress also degraded ‘Suppression of Stims’, as stimming was one means for people to comfort/regulate themselves.^25^ Camouflaging in and of itself could be straining, and one participant noted: ‘When I have had to do a lot of camouflaging in a high stress environment, I feel as though I’ve lost track of who I really am, and that my actual self is floating somewhere above me like a balloon’.^13^ This leads to the next point.

#### Theme 4: duration of camouflaging

There was wide variation in the extent of camouflaging.^13^ Some individuals camouflaged (in an amorphous sense) ‘all the time’,^4^ by ‘constantly’ monitoring themselves.^9^ In terms of ‘Performativity’, one person described ‘act[ing] every single waking moment’.^3^ In this regard, some individuals never experienced a space in which they felt safe to drop their camouflaging, even with family or friends – and constant camouflaging also signified the constant felt *need* to. For others, owing to the amount of stress and strain they experienced, lengthy spans of camouflaging were hard to maintain.^13^ Cognitive strategies could be especially difficult to sustain,^17^ with people having to push through the evening^24^ or the half hour appointment.^22^

## Discussion

Our integrative review consolidated conceptions of camouflaging strategies and related contextual moderators, to serve as a *tentative* framework (Gheondea-Eladi, 2015; Grant & Booth, 2009). The demographics of the sample on which this framework was built are reflected in Table 2. As with Cook, Hull, et al.’s (2021) review, roughly two-thirds of the reported sample was female, suggesting gendered self-selection. This aligns with studies reflecting more late-diagnosed women with (above-)average intelligence (Cook, Hull, et al., 2021; Libsack et al., 2021; Zhuang et al., 2023). Similar to Libsack et al.’s (2021) review, only around 20% of ethnic or racial data is reported, with roughly 90% of reported samples being White (Cook, Hull, et al., 2021).

**Table 2.** Demographic details.

	Total/2669	Reported total	Comment
Age	- -	Range: 15-79 years Mean: 35.40 years	- Range and Mean are incongruent (i.e. based on different subsets of data). Data was not skewed by high-school or late-life-stage studies
Gender	Female: 39.68% Male: 19.52% Non-binary: 3.86% Unknown: 36.79%	Female: 62.77% Male: 30.88% Non-binary: 6.11%	- Gender versus natal sex was not systematically delineated in the data. - Even when female-only studies are excluded, only 34.19% of men still comprise the sample.
Race	White: 17.87% Mixed Race: 1.09% Asian: 0.30% Black: 0.04% Hispanic: 0.04% Unknown: 80.67%	White: 92.44% Mixed Race: 5.62% Asian: 1.55% Black: 0.19% Hispanic: 0.19%	- Mixed-race included 1 partly aboriginal individual. - The 1 reported black individual was black-British
Education	< Secondary: 1.35% Secondary: 17.16% Tertiary: 11.20% Unknown: 70.29%	< Secondary: 4.54% Secondary: 57.76% Tertiary: 37.70%	- Tertiary includes university & trades training - At least 6.56% of the sample was busy studying in some capacity.
Employment	Busy Studying or Working: 24.02% Unemployed/Unable to Work: 8.13% Other: 2.54% Unknown: 65.31%	Busy Studying or Working: 69.22% Unemployed/Unable to Work: 25.20% Other: 7.34%	‘Other’ includes retirees, full-time carers or volunteer workers.

*Note.* For nationality, the majority of participants came from the United Kingdom (33.01%), with Australia and New Zealand (combined) coming in second at 4.35%. These data were not provided for 57.06% of the sample. Economic/living circumstance was too sparsely reported to derive results.

### Strategies

**Figure 2.**
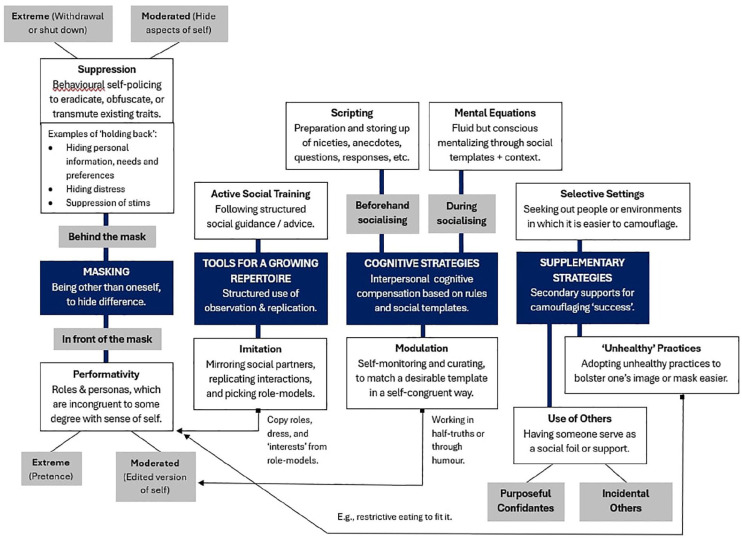
Summary of camouflaging strategies per the Results section. *Note.* Imitation, Modulation, and Unhealthy Practices all had examples of overlap with Performativity, reflected by the black arrows.

Study Question 1 queried the types of camouflaging strategies that exist, with six inter-related themes found. **(1) Masking** (including Suppression and Performativity) reflected a dual process similar to Ai et al.’s (2022) Goffman-aligned discussion of ‘backstage’ concealment of self that supports ‘frontstage’ self-representation. At the extreme, suppression reflects withdrawal, self-policing (e.g. of stimming; Collis et al., 2022) or ‘innocuous engagement’ (Cook et al., 2022) that keeps the individual safe, while performativity reflects adopting identity-incongruent personas. Within **(2) Tools for a Growing Repertoire** (including Imitation and Active Social Training), mirroring of others allowed for immediate assimilation, and observations and active studying of social patterns also allowed one to develop a repertoire of social templates to be imitated at a later point. **(3) Cognitive Strategies** (including Scripting, Modulation and Mental Equations) then reflected the use of such templates to thoughtfully navigate social encounters in neurotypical spaces, per what Livingston and Happé (2017) describe as ‘cognitive compensations’. **(4) Use of Others**, **(5) Unhealthy Practices** and **(6) Selective Settings** appear to supplement the effectiveness of other strategies (*discussed later*). It is notable that strategies across these domains, such as performativity, mirroring, modulation, scripting, use of others and selective settings, all fall under the umbrella of what has broadly been described as ‘Assimilation’ (e.g. Hull et al., 2017; i.e. social integration or blending in). While falling under a different topic (i.e. *motivations* to camouflage), we noted how stigma, negative feedback and dictates from others formed a crucial reason for the development of camouflaging as a self-protective mechanism. The progression and relationality of the abovementioned camouflaging strategies are visualised in Figure 2.

### Templating

One significant insight was the role that mimicry or delayed imitation played in scaffolding camouflaging strategies. This is because they iteratively helped develop social templates through a process of ‘doing’, assessing and refining. This thus afforded people more than just basic behavioural suppression. Per a Bayesian learning model, an individual infers cause-and-effect phenomena in an interaction. They then abstract these experiences into an increasingly flexible, generalist framework or top-down prediction (Amoruso et al., 2019; Gopnik & Wellman, 2012). In the context of social camouflaging, observational learning and instruction replace or supplement behaviours (e.g. transmuting stims) (Hickey et al., 2018; Kapp et al., 2019). Delayed replication of ‘successful’ social interactions, trialling different facets and repetition all help consolidate conceived social patterns into learned templates and heuristics (Leedham et al., 2020).

If we postulate a progression through the strategies, **suppressed** behaviours may be masked by **imitation** and **performativity**. **Imitation** builds social templates and thus **scripts** to be trialled in iterative cycles. A consciously planned and desirable ‘output’ is prototyped (e.g. Cook, Crane, et al., 2021) as a self-**modulation** or role **persona**, often attached to a context. More complex templates allow more immediate social decoding (e.g. **mental equations**) (Dachez & Ndobo, 2018; Forster & Pearson, 2020) which improve prediction and thus compensation. The implication is that imitative processes or motivations – and conscious, top-down self-training – should be given greater weight when assessing for autism. This means investigating experiential facets of camouflaging and the conscious efforts involved, rather than just one’s external presentation.

### Contextual moderators

Study Question 2 queried the contextual factors that moderate camouflaging strategies, with four related themes found: **(1) Unclear and Unexpected Cues**, **(2) Number of People**, **(3) Distress and Overwhelm** and **(4) Duration of Camouflaging**. Respectively – unpredictability, an increase in the number of social partners, strain and fatigue, and extended periods of interacting would all have a deleterious effect on an individual’s capacity to camouflage. Indeed, crowded, unstructured spaces might increase strain, revealing one’s true distress level, and possibly ramp up the need for comforting self-stimulation (Davidson & Henderson, 2010). The supplementary strategies of Use of Others, Unhealthy Practices and Selective Settings appeared to interplay with contextual factors to improve camouflaging efforts. For example, trusted others could serve as social foils that spearhead engagement and diminish the impact of crowds. They could likewise explain unclear cues, helping the autistic individual arrive at the appropriate template to draw on. Selectively choosing, for example, calmer and quieter spaces helped defend against sensory assaults that would ramp up the individual’s distress, and likewise practices such as drinking could reign in a certain degree of anxiety (Bradley et al., 2021).

### Strategy complexity

The deleterious impact of contextual factors would also depend on the level of complexity the individual can manage, as context impacts on the predictive use of cognitive templates (Crompton et al., 2020). There is variability in how complex one’s strategies develop based on personal capacities, motivation, self-insight (Hull & Mandy, 2021), executive functioning (Ai et al., 2022) or maturation (Livingston & Happé, 2017). To start with; predictions developed through Bayesian learning are impacted by the use of ‘priors’ (i.e. prior data that is drawn/built-on) (e.g. Ai et al., 2022; Amoruso et al., 2019). We distinguish *structural*-priors (broader, default expectations of a situation) and *contextual*-priors (fluid, short-term cues that shift predictions; Amoruso et al., 2019). Contextual priors may especially hamper predictability in autistic socialising. Thus, situations that place greater emphasis on the use of contextual priors may deteriorate camouflaging efforts more quickly.

For example, basic templating (e.g. **scripts**) appears to implicate structural priors – which inform a default response-set. Yet, this may falter due to inflexibility (Livingston, Shah, & Happé, 2019) when presented with shifting, unclear, unexpected cues. **Mental equations** would thus be the most complex tasks as they would make demands on use of structural *and* contextual priors. For a more comprehensive outline of Bayesian theory and computational modelling of impression management in autism, we recommend the article by Ai et al. (2022).

The implication of the above is that, in contexts that make high interpretive demands, the most flexibility is offered by the development of a nuanced ToM. The use of **mental equations** proves the capacity for ToM in autism, even if not evenly developed across all individuals. Rather than ToM serving as a marker for the presence or absence of autism, we could once again rather look to experiential input. For clinicians – individuals may present as superficially, diagnostically subclinical or unimpaired. Nonetheless, their subjective experience of distress and strain while trying to navigate environments of high interpretive demand may serve as its own clinical signpost for deeper inspection. Furthermore, it has been noted that camouflaging may negatively impact mental health (Khudiakova et al., 2024). It seems self-evident that autistic individuals may get caught in a lose-lose process. (a) Those unable to achieve sufficient strategy complexity suffer additive strain due to constant self-monitoring, while nonetheless remaining vulnerable to discrimination due to ‘ineffective’ masking. (b) Those who *are* able to achieve high strategy complexity may suffer exhaustion and possible burnout at trying to maintain compensation across varied, chaotic and extended contexts of interacting. This spectrum of different cons and ‘costs’ across camouflaging strategies might account for why reports on camouflaging outcomes appear inconsistent in the literature (Khudiakova et al., 2024).

### Limitations

#### Community involvement

The study was not developed with or reviewed with any autistic community collaboration, and as such, represents a weakness in terms of providing an external lens to describe experiences internal and deeply personal to those that mask.

#### Conscious camouflaging

Qualitative studies tend towards a self-reflection approach (Pearson & Rose, 2021). This means unconscious behaviours may not be well reflected in the results. It also means the sample is skewed, as studies exclude those who cannot self-reflect.

#### (In)formal diagnosis

Not all participants reflected confirmed diagnoses, and thus, their autistic-status is debatable. Yet, if camouflaging promotes neurotypical behaviour, those most adept at it may fall below diagnostic thresholds (Livingston, Colvert, et al., 2019).

#### Demographics

The *reported* sample was mostly White, British, female, and relatively educated. However, different (sub-)cultural groups may hold different expectations of what is socially acceptable and report variable degrees of autism detection (Matson et al., 2017). This is compounded by limited inclusion of people of colour in research, and overlapped experiences of ethnic discrimination (Jones et al., 2020). The number of UK participants raises questions of culturally modulated camouflaging, and so extension to other autistic groups remains theoretical.

#### Scope

We focused only on studies explicitly discussing camouflaging, or at least hiding aspects of oneself, with some depth. As such, we did not included studies that merely alluded to, or only briefly referenced, camouflaging (e.g. before the concept became mainstream).

### Implications

We have synthesised conceptions of autistic adult camouflaging into an amalgamated framework for the *strategies* used. Our contribution was thus to step beyond presumed agreement or narrative summation of the existing literature. By way of thematic meta-synthesis, we were able to confirm the breadth, consistency and agreement of themes across articles. Any inconsistencies between studies were reconcilable once contextual factors were considered.

#### For research and screening

One study goal was the need for conceptual clarity. The current framework provides one congruent conception of camouflaging to further research and delineates clear variables and testable assertions (such as the process of imitating, replicating and generalising social templates for context-specific use). Our recommendations include the following:

• We have posited six umbrella strategy types, and further subtypes, which we recommend be used to expand screening tool domains or exemplars (e.g. per Table 3), towards improving detection. Given a lack of demographic diversity in existing studies, it is recommended that items be further adapted for their relevant cultural milieus.
• Likewise, it is suggested that further investigation be directed towards understanding intentional imitative processes, and how these form a key, *reportable* experiential component of camouflaging in autism.
• Furthermore, historically quantitative camouflaging research has (partly) operationalised ‘true’ degree of autism by the level of ToM deficit present (Cook, Hull, et al., 2021; Hull et al., 2020). The implications of sophisticated cognitive templating and compensatory strategies is that some autistic individuals will not reliably be defined by such ToM measures, and as such, alternative means of establishing ‘baselines’ for such studies will need to be explored.

**Table 3.** Examples of camouflaging strategies per theme.

Suppression	Performativity
• Keeping quiet or speaking minimally in interactions • Suppressing innate behaviours or aspects of oneself • Minimise non-conventional interests/activities • Withholding feelings or signs of overwhelm • Minimise restricted repetitive, behaviours/OR/active self-monitoring and substitution of stims. • Avoidance of social contact (going under the radar) • Non-disclosure of personal information/preferences	• Pretending to be a specific, different person • Developing a context-specific persona(s) or identity variation – for example, the ‘good student’ • Pretending to be interested in a topic/faking interests • Using items of clothing to uphold a persona • Taking on a blasé or nonchalant air • Using socially accepted ailments (e.g. headache) to excuse social strain
Imitation	
• Mirroring other’s speech patterns (inc. accents), mannerisms, and body language during an interaction • Repeating the phrasing of others, copying their interests, and dressing like others/the group • After the interaction, acting out, re-enacting or practicing the behaviour of others (e.g. their style laughter). • Copying phrases and behaviours from different media sources, including from fictional characters.	
(General) Cognitive Strategies	
	Modulation
• Allocating specific facial expressions/behaviours to specific contexts/OR/matching rules and contexts • Using signs of interest (nodding, agreeing) to prompt the other person to keep speaking • Using direct questions (and ‘you’ statements) to prompt the other person to keep speaking • Setting an internal ‘reminder’ or ‘timer’ for when to stop speaking – minimising one’s own speaking time • Making general rather than specific statements • Responding to demarcated prompts (e.g. joke cues) • Pre-planning topics of conversation and responses • Creating sub-patterns of behaviour for each person • Using digital communication to allow extra time to respond	• Adjusting behaviours to a specific idea of what the public would consider the ‘good’ or ‘right’ way • Emphasising competence, intelligence or skills • Displaying positive attributes that others would see as ‘similar’, friendly, funny or nice • Selectively sharing normative interests and characteristics • Smoothening out tone and rhythm of one’s voice • Making direct/approximate eye contact with others • Over-emphasising responses so as to not appear ‘flat’ (displaying interest and emotional responsiveness) • Making use of humour to play off difficulties
Use of others	
• Keeping close to a sociable other, to appear sociable oneself (either subtly or intentionally having them lead) • Having a confidante available to explain unclear aspects of a situation, or to check written communication • Pretending to want company on an outing so that an individual is available to help with tasks	
‘Unhealthy’ practices	
• Using substances to feel less anxious and thus be ‘freed up’ to mask better • Using actual or faked use of substances to excuse faux pas in interactions • Adopting practices such as restrictive eating to fit in with certain social circles (i.e. do as others are doing)	
Selective settings	
• Moving somewhere else to start anew and ‘reinvent’ oneself (sometimes in a foreign country) • Findings spaces and places where community norms result in easier, slower or more straight forward socialising • Seeking out structured spaces with clear topics of focus, or structured social engagement (e.g. book clubs) – which might play into one’s personal strengths or be built around personal interests • Engaging people of a different nationality, culture, age range, etc. – so as to avoid direct social comparison • Befriend others who are also in some way socially excluded or vulnerable	

#### For practice and policy

Given the mental health risks connected to camouflaging, improved detection of autistic individuals by healthcare workers is necessary, for which we provide a list of examples (see Table 3). Clinicians are advised to make use of the framework to improve screening, think through therapeutic pathways and provide crucial psychoeducation to clients. Clinicians are also advised to step beyond mere observation of the presence or absence of autism-associated symptoms, towards also spotting *performance* of neurotypicality. One aspect of this raised in our discussion is the fact that ToM cannot be used as a definitive marker for autism. Instead, subjective reports of effort, strain or distress should be considered when deciding whether outwardly ‘passable’ (neurotypical-seeming) behaviour is actually a reflection of camouflaging and thus social integrative difficulties. Clinicians would likewise take into account the manner and reasoning for how the individual engages in different spaces, spotting variation across contexts, and not overly stereotyping how autism will present.

## Conclusion

We conducted an integrative systematic review towards consolidating camouflaging strategies in autistic adults and thereby achieved our goals of attaining conceptual clarity, confirming agreement across the literature and providing a listing of such strategies. We found six inter-related themes of camouflaging, with a spotlight on the role of *imitation* as not just a strategy, but also an entire learning structure within which the individual grows their repertoire of social templates. Furthermore, four strategy-moderating contextual factors were found. However, most study participants come from the United Kingdom, raising a question of the degree to which the current conceptions of camouflaging are culturally modulated. As such, further research into contextual and cultural influences is recommended. The results further encourage future quantitative research to concretely place measures of camouflaging within this (or other) frameworks, for consistency in tracking camouflaging behaviours.

## Supplemental Material

sj-docx-1-aut-10.1177_13623613251335472 – Supplemental material for Consolidating a framework of autistic camouflaging strategies: An integrative systematic review Supplemental material, sj-docx-1-aut-10.1177_13623613251335472 for Consolidating a framework of autistic camouflaging strategies: An integrative systematic review by Jacques Nel, Maxine Spedding and Susan Malcolm-Smith in Autism
