# Supplementary material for: Consolidating a framework of autistic camouflaging strategies: An integrative systematic review
Source: Autism. 2025 May 30;29(10):2379–94. doi: 10.1177/13623613251335472 (PMC12417612; doi:10.1177/13623613251335472)
Supplement: sj-docx-1-aut-10.1177_13623613251335472 – Supplemental material for Consolidating a framework of autistic camouflaging strategies: An integrative systematic review [file sj-docx-1-aut-10.1177_13623613251335472.docx]

# Appendix 1: Database Search Strings

| ***EBSCOhost, Web of Science and Scopus*** | ***PubMED Search*** |
| --- | --- |
| **Search string**: ("Autism Spectrum Disorder" OR "Autism Spectrum Disorder" OR "Autism Spectrum Disorders" OR "Asperger Syndrome" OR "Aspergers Syndrome" OR "Autistic Disorder" OR ASD OR ASC OR autism) AND (Camouflage OR Camouflaging OR masking OR compensate OR compensating OR imitate OR imitating OR mimic OR mimicking OR copy OR copying OR hide OR hiding OR passing OR pass) AND (adult OR adulthood OR adolescent OR adolescents OR adolescence OR adults) | **Search string**: ((((((((((((("Autism Spectrum Disorder"[Mesh]) OR ("Child Development Disorders, Pervasive"[Mesh])) OR ("Asperger Syndrome"[Mesh])) OR ("Autistic Disorder"[Mesh])) OR ("Autism Spectrum Disorder")) OR ("Autism Spectrum Disorders")) OR ("Asperger Syndrome")) OR ("Asperger's Syndrome")) OR ("Autistic Disorder")) OR (ASD)) OR (ASC)) OR (autism)) AND ((((((((((((((("Camouflage") OR ("Camouflaging")) OR ("masking")) OR ("compensate")) OR ("compensating")) OR ("imitate")) OR ("imitating")) OR ("mimic")) OR ("mimicking")) OR ("copy")) OR ("copying")) OR ("hide")) OR ("hiding")) OR ("passing")) OR ("pass"))) AND ((((((((("Adult"[Mesh]) OR ("Young Adult"[Mesh])) OR ("Adolescent"[Mesh])) OR (adult)) OR ("adulthood")) OR (adolescent)) OR (adolescents)) OR ("adolescence")) OR ("adults")) |
| **Filters across databases**: No filters applied  **Limiters across databases**: Language – English (***all databases**)  Source Type – Academic Journals (***EBSCOhost**) | |

# Appendix 2: Quality Assessment and Article Scores

## Section 1: Scoring Protocol

The Lorenc et al. (2014), protocol-adjusted version of the Hawker et al. assessment framework (2002, as cited in Lorenc et al., 2014, Appendix 5) includes a numerical scoring and cut-off system. Quality per each item is assigned a value ranged between ‘very poor’ (1), ‘poor’ (2), ‘fair’ (3) and ‘good’ (4). Thus, across 9 items, the minimum score possible is 9 and the highest is 36. Lorenc et al. (2014) delineated cut-off Grades for the overall papers as being High Quality [A] (30-36 points), Medium Quality [B] (24-29 points) and Low Quality [C] (9-23 points).

Adaptations were made in the protocol stage to accommodate for the inclusion of case studies. As no case studies were ultimately included, the original framework is presented below. Additionally, I added to the proposed scoring grid (as below in blue) to more clearly operationalise the scoring criteria. Original scoring cut-off points were retained.

| 1. | ***Abstract and title*: Did they provide a clear description of the study?**  **[Factors: 1) Clear Background/Rationale, 2) Clear aim(s), 3) Clear method AND sampling, 4) Clear and study-topic relevant outline of results]; and; title at minimum alerts to possibility of study relevance. **Qualitative consideration of the depth and usefulness of the information raised.**  **Good** Structured abstract with full information & clear title. **All 4 factors + somewhat relevant title.**  **Fair** Abstract with most of the information. **3 factors + somewhat relevant title, or all 4 factors + completely theme-unrelated title.**  **Poor** Inadequate abstract. **2 factors** **+ somewhat relevant title, or 3 factors + completely unrelated title.**  **Very Poor** No abstract. **1 factor** **+ somewhat relevant title, or 2 factors + completely unrelated title.** |
| --- | --- |
| 2. | ***Introduction and aims*: Was there a good background and clear statement of the aims of the research?**  **[Factors: *INTRODUCTION* 1) Clear and topic relevant literature, 2) gap in literature highlighted, and *AIMS* 3) clear aims and objectives, and 4) explicit research questions or hypotheses]**  **Good** Full but concise background to discussion/study containing up-to-date literature review and highlighting gaps in knowledge. Clear statement of aim **AND** objectives including research questions **[i.e., AIMS: Both 1) aims and 2) research questions/hypotheses provided] [All 4 factors present]**  **Fair** Some background and literature review. Research questions **OR aims outlined**. **[3 factors present]**  **Poor** Some background but no aim / objectives / questions, OR aims /objectives but inadequate background.  **Very Poor** No mention of aims/objectives. No background or literature review. |
| 3. | ***Method and data*: Is the method appropriate and clearly explained?**  **[Factors: *METHOD* 1) Clear and appropriate qualitative method, 2) Survey or interview questions provided, and *DATA* 3) description of data [including collection or interview methods, 4) data recording is clear]; and; Qualitatively: considerations around the depth or appropriateness of discussion.**  ***Text analyses must be assessed on their own merit.**  **Good** Method is appropriate and described clearly (e.g., questionnaires included). Clear details of the data collection and recording. **[All 4 factors presented with sufficient depth]**  **Fair** Method appropriate, description could be better. Data described. **[3 factors presented with sufficient depth, or 3+ factors only vaguely outlined]. Data description MUST be an included factor.**  **Poor** Questionable whether method is appropriate **AND/OR** Method described inadequately **AND/OR** Little description of data.  **Very Poor** No mention of method, AND/OR Method inappropriate, AND/OR No details of data. |
| 4. | ***Sampling***: **Was the sampling strategy appropriate to address the aims?**  **[Factors: *METHODOLOGICALLY*: 1) appropriate sample size, 2) sample rationale and 3) recruitment routes are clear, and *DEMOGRAPHICALLY*: 4) age, 5) sex/gender identity, 6) ethnicity and/or nationality, and 7) socio-economic circumstance, are all clear]; and; qualitative considerations (factors must be apparent for the relevant total or subset of participants).**  **Good** Details (age/gender/race/context) of who was studied and how they were recruited. Why this group was targeted. The sample size was justified for the study. Response rates shown and explained. **[All methodological factors included and at least 3 demographic factors]**  **Fair** Sample size justified. Most information given, but some missing. **[At least 2 factors of each domain provided]**  **Poor** Sampling mentioned but few descriptive details. **[At least 2 factors present]** **Very Poor** No details of sample. |
| 5. | ***Data analysis*: Was the description of the data analysis sufficiently rigorous?**  **[Possible factors: 1) Style of analysis (e.g., thematic, discourse, etc.), 2) analytic steps including who were the principle coders, 3) evidence of themes being audited (e.g., supervisors, sample respondent validation, etc.), 4) analytic framework and theoretical underpinning (e.g., inductive, critical realism, social constructionism].**  **Good** Clear description of how analysis was done. Qualitative: Description of how themes derived/ respondent validation or triangulation. **[All 4 factors present]**  **Fair** Qualitative: Descriptive discussion of analysis. **[3 factors present with style of analysis being mandatory]**  **Poor** Minimal details about analysis. **[2 factors present with style of analysis being mandatory]**  **Very Poor** No discussion of analysis. **[1 or less factor present]** |
| 6. | ***Ethics and bias*: Have ethical issues been addressed, and what has necessary ethical approval gained? Has the relationship between researchers and participants been adequately considered?**  **[Factors: *ETHICS* 1) Ethical clearance or outline of research practice guidelines is given, 2) consent procedures are addressed, 3) data privacy or secure management is addressed, and *BIAS* 4) the researcher can position themselves and their possible bias in relation to results and sample];**  **Good** Ethics: Where necessary issues of confidentiality, sensitivity, and consent were addressed. **Ethical basis must be discussed.** Bias: Researcher was reflexive and/or aware of own bias. **[All 4 factors present]**  **Fair** Lip service was paid to above (i.e., **both ethical and bias** issues were acknowledged **vaguely**), **or one domain is discussed in-depth whilst the other is absent.**  **Poor** Brief mention of issues. **[Only 1 domain discussed briefly in vague detail]**  **Very Poor** No mention of issues. **No domains discussed.** |
| 7. | ***Results*: Is there a clear statement of the findings?**  **[Factors: 1) Results/themes are clear and logical in their layout and discussion, 2) text and tables/figure models are structured similarly and explained by text (de facto awarded point if no tables are present), 3) results meet study aims or study focus, 4) sufficient justificatory data provided]**  **Good** Findings explicit, easy to understand, and in logical progression. Tables, if present, are explained in text. Results relate directly to aims. Sufficient data are presented to support findings. **[All 4 factors present]**  **Fair** Findings mentioned but more explanation could be given. Data presented relate directly to results. **[3 factors present]**  **Poor** Findings presented haphazardly, not explained, and do not progress logically from results. **[2 factors present]**  **Very Poor** Findings not mentioned or do not relate to aims. |
| 8. | ***Transferability or generalizabilit*y: Are the findings of this study transferable (generalizable) to a wider population?**  **The core unified consideration will be whether sufficient details are provided to allow for extrapolation of the data either more broadly (generalisability) or laterally (to other populations or sample groups). This incorporates understanding how the person may be placed in relation to context or setting (e.g., racial, cultural or sexuality status, economic status, living circumstance, nationality, education and occupation level, etc.]**  **Good** Context and setting of the study is described sufficiently to allow comparison with other contexts and settings, plus high score in Question 4 (sampling).  **Fair** Some context and setting described, but more needed to replicate or compare the study with others, PLUS fair score or higher in Question 4.  **Poor** Minimal description of context/setting.  **Very Poor** No description of context/setting. |
| 9. | ***Implications and usefulness:* How important are these findings to policy and practice?**  **[Factors: 1) the novelty or contribution of the study is clear; 2) 1 or more clear recommendations for future research (thematically or methodologically); 3) 1 or more clear recommendations for application to practice or policy]**  **Good** Contributes something new and/or different in terms of understanding/insight or perspective. Suggests ideas for further research. Suggests implications for policy and/or practice. **[All 3 factors present]**  **Fair** Two of the above (state what is missing in comments).  **Poor** Only one of the above.  **Very Poor** None of the above. |

Please contact the authors for access to the quality assessment of a specific article included in the study (nel.jacques@outlook.com)

# Appendix 3: Data Tabulation

| **Section 1: Basic Sample Demographics** | | | | **Gender**  Female F Male M Other O | **Ethnicity or Culture** | **Employment**  Student S; Full-time FT; Part-time PT; Volunteer V; Retired R; Jobless J | **Education level**  Secondary Studies S,  Tertiary Studies TS  Unqualified U |
| --- | --- | --- | --- | --- | --- | --- | --- |
| **Study** | **Sample Size** (attrition/ exclusion)  [total] | **Nationality or Milieu** | **Age range** (rounded mean) |  |  |  |  |
| Baldwin & Costley (2015) | 82 | AU (*implied natal + migrant*) | 18-64 (32.7) | 82 F | c. 56 ‘AU’ (68%); c. 15 ‘UK Or Eu’ (18%); c. 11 ‘Other’(14%) | c. 14 S (17%) c. 20 FT (40%); c. 31 PT (60%); **c. 17 Unknown (21%)** | c. 15 U (18%) (presumed);  c. 60 S (89%)  c. 7 TS (11%) |
| Bargiela et al. (2016) | 14 (3) | UK | 19-30 (26.7) | 14 F | *Not*  *provided* | 3 S; 7 FT;  3 V; 1 Carer | *Not*  *provided* |
| Bernadin et al. (2021) | **4** **age-relevant** [ASD group; 10] | US | 16-18 (17.0) | 3 F, 1 M | *Not*  *provided* | 4 S (*high-schoolers*) | Presumed mainstream highschooler |
| Botha et al. (2020) | 20 (2) | **1** S. America; **14** UK; ***5*** *“Europe, America, Israel or New Zealand”* | 21-62 (37.2) | 9 F, 9 M,  2 O | 13 White-UK; 1 Black-UK; 5 White non-UK; 1 Mixed Race (S American) | *Not*  *provided* | *Not*  *provided* |
| Bradley et al. (2021) | 277 (69) | UK | **18+** (38.7) | 184 F, 93M | *Not*  *provided* | 32 S; 147 Employed;  16 V; 74 J; 5 R;  **3 Unspecified** | 268 S (minimum?)  9 Special Needs Education |
| Cage et al. (2018) | **63** [**111**] | UK | 18-72 (36.4)  From Total | **From Total:**  60 F, 27 M  14 O  **10 Unknown** | **From Total:**  70 White-UK 18 White Non-UK;  4 Mixed Race; 1 Asian  7 Other *reported  **11 Unknown** | **From Total:**  16 S; 18 FT;  12 PT; 7 Self-Employed;  33 J; 5 R;  2 Other *reported;  **18 Unknown** | **From Total:**  4 U; 27 S (Presumed) Undergraduate or Trades Training; 27 Postgraduate;  12 Other *reported;  **9 Unknown** |
| Cage & Troxell-Whitman (2019) | **91** [**262**] | **//** UK? (Based on prior study) | 18-66 (33.6)  Total sample | **From Total:**  135 F;  111 M; 12 O  4 Un-disclosed | **From Total:**  c. 225 White (85.8%); c. 22 Mixed Race (8.4%); c. 7 Asian (2.7%); **c. 8 Unknown or Undisclosed (3%)** | **From Total:**  c. 20 Self-employed; c. 63 S (23.8%); c 76 FT (29%); c. 28 PT (10.4%); (7.7%); c. 59 J (22.3%); c. 3 R (1.2%); c. 10 Carer (3.8%); **c. 3 Undisclose (1.2%)** | **From Total:**  c. 16 U (6.1%)  c. 63 S (Presumed) (23.8); c. 78 Under-graduate or Trades Training (29.8%); c. 65 Postgraduate (24.9%); **c. 40 Unknown / Un-disclosed (15.4%)** |
| Collis et al. (2022) | 12 | **//** UK (presumed by in-person interviews) | 18-23 (20.6) | 4 F, 5 M,  3 O | *Not*  *provided* | 10 S; 1 FT; 1 J | 10 S; 2 Under-graduate (complete) |
| Cook et al. (2018) | **4 age-relevant**, [11] *Teen + parent = 1 unit | UK | 16-17 (16.3) | 4 age relevant F | 4 White-UK | 4 S (*high-schoolers*) | 2 High School (mainstream); 2 Special Needs Edu. |
| Cook et al. (2021) | 17 (5) | UK | 20-64 (44.5) | 8 F, 6 M,  3 O | 12 White-UK  3 White Non-UK; 1 Mixed-Race Non-UK; 1 Hispanic | 4 S; 6 FT; 7 PT;  2 V; 1 Carer;  1 Unknown **[Not mutually exclusive]** | 1 S; 8 TS |
| Crompton et al (2020) | 12 | UK | 21-51 (33.6) | 10 F, 2 M | *Not*  *Provided* | *Not*  *provided* | *Average 18 years of edu.(thus: majority at a post-graduate level ? ) |
| Dachez & Ndobo (2018) | 31 | France | 15-53 (31.7) | 17 F, 14 M | *Not*  *provided* | c. 1 S (high school) (3%); c. 7 S (uni) 23%); c.5 Employed (21% of c. 23 remainder); **c. 18 Unspecified (of c. 23 non-student remainder)** | c. 1 High school (3%); c. 6 S (Presumed); c. 24 TS (74% of subset (17) + ongoing students) |
| Davidson & Henderson (2010) | 45 | *Not*  *provided* | *Not*  *provided* | *Not*  *provided* | *Not*  *Provided* | *Not*  *provided* | *Not*  *Provided* |
| Forster & Pearson (2020) | 5 | **//** UK? (based on method /location) | 22-25 (22.6) | 2 F, 3 M | *Not*  *Provided* | *Not*  *provided* | *Not*  *provided* |
| Harmens et al. (2022) | 24 | **6** UK; **16** USA;  **1** AU; **1** Canada | **18+** (//) | 24 F | Authors *believe* most were white | *Not*  *provided* | *Not*  *provided* |
| Hickey et al. (2018) | 13 | UK | 51-71 (60.4) | 3 F, 10 M | 11 White-UK  2 White Non-UK | 2 FT; 3 PT; 4 R, 4 J (>10 years) | 5 S; 6 Under-graduate; 1 Post-graduate; 1 Uni-versity (unspecified) |
| Howard & Sedgewick (2021) | 245 (unclear if all gave quali. input) | UK | 16-74 (40.4) | 151 F, 61 M, 33 O | *Not*  *provided* | *Not*  *provided* | *Not*  *provided* |
| Hull et al. (2017) | 92 (9) | **51** UK; **16** N. America; **15** W. Europe; **10** Other | 18-79 (43.2) | 55 F, 2 M,  7 O | *Not*  *provided* | *Not*  *provided* | *Not*  *provided* |
| Kapp et al. (2018) | 31 | UK | 21-56 (//) | 10 F, 20 M,  1 O | *Not*  *provided* | 5 S; 3 V; 16 J  7 Employed | *Not*  *provided* |
| Leedham et al. (2020) | 11 | UK | 43-64 (50.8) | 11 F | *Not*  *provided* | *Not*  *provided* | *Not*  *provided* |
| Lilley et al. (2021) | 26 (2) | AU | 45-72 (52.9) | 14 F, 10 M, 2 O | 25 White AU; 1 Mixed AU-Aboriginal | 19 Employed  1 V; 5 J; 1 R | 1 U; 1 S; 16 Under-graduate; 8 Post-graduate |
| Livingston et al. (2019) | **77 in ASD group** | **11** N. America; **53** UK; **6** Europe **6** AU; **1** Africa | 18-77 (36.6) | 46 F, 21 M,  10 O | *Not*  *provided* | 16 S; 30 FT;  12 PT; 4 V; 15 J. | *Not*  *provided* |
| Mantzalas et al. (2022) | 683 | **//** International ? (Social media) | Approx. 18-76 | 61 F (min)  60 M (min)  1 Unknown | *Not*  *provided* | *Not*  *provided* | *Not*  *provided* |
| Miller et al. (2021) | **144 in ASD group** (106) | **//** International ? | 18-74 (36.3) | 101 F, 28 M,  15 O | *Not*  *provided* | *Not*  *provided* | *Not*  *provided* |
| Milner et al. (2019) | **22 inc. 4 parents** | UK | Unclear (16-18+ ?) at time of study | 18 F  +Additional 4 parents | *Not*  *provided* | *Not*  *provided* | *Not*  *provided* |
| Milton & Sims (2016) | 361 | Magazine source is both UK-based & International | Unclear (for adults readerships) | *Not*  *provided* | *Not*  *provided* | *Not*  *provided* | *Not*  *provided* |
| Schneid & Raz (2020) | 24 | Israel | 16-55 (31.0) | 13 F, 10 M,  1 O | Implied to be Israeli, but no further details | 8 Employed; 11 V; 4 J;  1 Unknown | *Not*  *provided* |
| Tint & Weiss (2018) | 20 | Canada | 19-69 (35.5) | 20 F | 18 White 90%)  2 Other 10%) | 11 FT (school or employment) (55%); 2 PT (school or employment) (10%); 1 R; (5%); 6 Other (30%) | **Already Attained***  4 S (20%);  13 Under-graduate or Trade Training (65%); 3 Post-graduate (15%) |

| **SUMMARY** | | | | | | |
| --- | --- | --- | --- | --- | --- | --- |
| **Sample Size** | **Nationality or Milieu** | **Age Range** | **Gender** | **Ethnicity or Culture** | **Employment** | **Education level** |
| **MAXIMUM**:  2669 participants | **1523 unclear**  **minimum: 881** **UK**  **[Suspected: 1160 UK]**  **minimum 115 Australia**  **minimum 69 “North America”**  Canada 21  United States 20  Unclear 28 [Possible 1 extra]  **minimum 53 “Europe”**  West EU 46 -(France 31)  Unclear 7 [Possible 1 extra]  **minimum 25 Israel** [Possible 1 extra]  **minimum 1 “Africa”**  **minimum 1 “South America”**  **minimum 1 New Zealand** [Possible 1 extra] | **Range**:  15 – (min) 79;  729 Data pot provided  **Mean age from available data: 35.40**  *Not provided for 1166 participants | 4 Parents (unspecified)  **1059 Women**  **521 Men**  **103 ‘Other’**  982 Unknown  [1524 have clear gender reporting in male-inclusive studies] | **2103 Unclear**  **Minimum 477 White**  White-UK,  White-AU,  And Other  **Minimum 29 Mixed**  **Minimum 8 Asian** *unspecified  **Minimum 1 Black-UK individual**  **Minimum 1 Hispanic individual** *unspecified | **1743 Unclear**  **Minimum 641 Full-time Occupied**  9 High School  166 Tertiary  154 Employed full  Time  86 Part-time  Employment  213 Employment  Unspecified (inc.  13 Self-employed)  **Minimum 19 retired**  **Minimum 217 unable to work or unemployed**  **Minimum 38 Volunteers**  **Minimum 11 Carers** | **1876 Unclear;**  **Minimum 36 Unqualified**  **Minimum 458 Secondary Studies**  447 Mainstream  (presumed)  11 Special Needs  (specified as such)  **Minimum 299 Tertiary Studies**  155  Undergraduate  112 Postgraduate  32 *Unclear* |

| **Study** | **Study Type** (Focus)  **Collection Method** (Source) | **Diagnostic Status: Autism** [Screen score range]  **No studies themselves did more than screen* | **IDD Status** [IQ score] | **Comorbidities** [Screen: Score Range] (SR = Self-Reported) |
| --- | --- | --- | --- | --- |
| Baldwin & Costley (2015) | **Mixed** (Qualitative)  **Survey** (Self-response) | 82 Formal diagnoses [//] | 82 Self-reported no IDD [//] | *“A minority alluded to […] (OCD)-type behaviours”* (SR); c. 70 Consider self to have a mental health condition, e.g., anxiety or depression (85%) – most commonly stress & anxiety (SR) |
| Bargiela et al. (2016) | **Mixed** (Qualitative)  **Survey and Single Interview** (Self-response) | 14 Formal diagnoses [AQ-10: 6-10] [Cut-off: 6] | 14 Self-reported and partially-screened, no IDD [WTAR Verbal IQ: 85-124] | 13 Anxiety [HADS-A: 7-20] [Cut-off: 8]  3 Depression [HADS-D: 1-15][Cut-off: 8] |
| Bernadin et al. (2021) | **Mixed** (Qualitative)  **Survey and Single Interview** (Self-response) | 4 Self-reported (formality *presumed* based off discussion of sample) [//] | 4 Self-reported no IDD [//]  (age-relevant group) | *Not*  *provided* |
| Botha et al. (2020) | **Qualitative** (//)  **Single Interview** (Self-response) | 15 Formal diagnoses; 5 Self-*identifying* [//]; Some individuals indicated as being non-verbal | *Not*  *Provided* | *Not*  *provided* |
| Bradley et al. (2021) | **Mixed** (Qualitative)  **Survey** (Self-response) | 206 Formal diagnoses (reported in-table) [AQ: mean 36.55]  [Cut-off: 26]  71 Self-*identifying* (reported in-table) [AQ: mean 33.55]  [Cut-off: 26] **(in-text only reports female figures)** | 277 no IDD [unclear how this was derived] | 211 Depression (SR); 185 Anxiety (SR)  44 OCD (SR); 19 Bipolar (SR); 32 PD (SR); 8 Schizophrenia (SR); 19 Anorexia N (SR); 8 Bulimia (SR); 22 Myalgic Encephalopathy (SR); 5 Tourette’s (SR); 10 Epilepsy (SR); 23 Dyspraxia (SR); 35 (S)LDs (SR); 20 ADHD (SR); 4 Developmental delay (SR) |
| Cage et al. (2018) | **Mixed** (Quantitative)  **Survey** (Self-response) | 100 Formal diagnoses (total sample)  11 Self-*identifying* (total sample) | *Not*  *provided* | 57 Depression (SR); 62 Anxiety (SR); 18 ADHD (SR); 35 Social Anxiety (SR); 18 OCD (SR); 9 PTSD (SR); 7 Bipolar (SR); 4 Tourette’s (SR)  [DASS-21 – Anxiety and Depression] |
| Cage & Troxell-Whitman (2019) | **Mixed** (Quantitative)  **Survey** (Self-response) | 262 Formal diagnoses (total sample)  [RAADS-14: 14-42]  [Cut-off: 14] | *Not*  *provided* | c. 136 Anxiety (51.9%) (SR); c. 38 ADHD (14.5%) (SR); c. 8 Bipolar (3.1%) (SR); c. 133 Depression (50.8%) (SR); c. 20 OCD (7.6%) (SR)  c. 25 PTSD (9.5%) (SR); c. 62 Social Anxiety (23.7%) (SR); c. 5 Tourette’s (1.9%) (SR)  [DASS-21 – Anxiety and Depression] |
| Collis et al. (2022) | **Mixed** (Qualitative)  **Survey and Single Interview** (Self-response) | 12 Formal diagnoses [//] | 12 no IDD (unclear how it was derived) | *Not*  *provided* |
| Cook et al. (2018) | **Qualitative** (//)  **Single Interview** (Triangulated: Parent-daughter dyads) | 4 Formal diagnoses [//] (age-relevant group] | *Not*  *provided* | 1 OCD (SR); 1 Anxiety (SR); Epilepsy (SR); 1 Depression (SR); 1 Learning Difficulties (SR); 1 Facial Tic Disorder |
| Cook et al. (2021) | **Qualitative** (//)  **Single Interview** (Self-response) | 17 Formal diagnoses  [AQ: Mean 39.71, **all** above cut-off 26] | 17 Self-reported and screened no IDD [TOPF: Mean 112.47, all > 70] | *Not*  *provided* |
| Crompton et al (2020) | **Mixed** (Qualitative)  **Single Interview** (Self-response) | 12 Formal diagnoses [AQ: 25-47] [Cut-off: 26] | 12 Self-reported and screened no IDD  [WASI-II: 99-140] | *Not*  *provided* |
| Dachez & Ndobo (2018) | **Qualitative** (//)  **Single Interview** (Self-response) | 31 Formal diagnoses [//] | 31 Self-reported no IDD [//] | *Not*  *provided* |
| Davidson & Henderson (2010) | **Qualitative** (//)  **Text Review** (Self-response) | 45 Self-reported (no further details) [//] | *Not*  *provided* | *Not*  *provided* |
| Forster & Pearson (2020) | **Qualitative** (//)  **Single Interview** (Self-response) | 5 Formal diagnoses [//] | *Not*  *provided* | *Not*  *provided* |
| Harmens et al. (2022) | **Qualitative** (//)  **Media Text Review** (Self-response) | c. 23 Formal diagnoses [//]  Minimum 1 Self-*identifying* [//] | *Not*  *provided* | 12 Depression (SR); 10 Anxiety (SR); 1 Bipolar (SR); 1 Suicidality (SR); 2 ADHD (SR); 1 Eating Disorder (SR); 1 PTSD (SR); 1 DID (SR) |
| Hickey et al. (2018) | **Mixed** (Qualitative)  **Single Interview** (Self-response) | 13 Formal diagnoses [AQ: 17-45, 3 below cut-off of 26] | 13 Self-reported no IDD [//] | 10 reported 1+ comorbidity  **12 (mild) Anxiety +** [HADS: 0-20]  **3 (mild) Depression +** [HADS: 0-16] |
| Howard & Sedgewick (2021) | **Mixed** (//)  **Survey** (Self-response) | 204 Formal diagnosis; 41 Self-*identifying* (including those still in the process of diagnosis) [AQ-10: **0**-10] [Cut-off: 6 ***not used**] (unclear if qualitative data came from the total no. participants) | *Was not checked | **Unclear Anxiety**  [GAD-7: Mean 11.56, 0-21]  [Min cut-off: 5] |
| Hull et al. (2017) | **Qualitative** (//)  **Survey** (Self-response) | 92 Formal diagnoses [//] | *Was not checked | *Not*  *provided* |
| Kapp et al. (2018) | **Qualitative** (//)  **Single & Group Interviews** (Self-response) | 31 Formal diagnoses [//] | *Not*  *provided* | *Not*  *Provided* |
| Leedham et al. (2020) | **Qualitative** (//)  **Survey** (Self-response) | 11 Formal diagnoses [//] | *Not*  *provided* | 1 Depression (SR); 3 ADHD (SR)  2 Learning difficulties or disorder (SR)  1 Epilepsy (SR); 1 Dyspraxia (SR)  1 PTSD (SR) |
| Lilley et al. (2021) | **Qualitative** (//)  **Single Interview** (Self-response) | 26 Formal diagnoses [//] | 26 Self-reported no IDD [//] | 12 Anxiety (SR); 3 ADHD (SR)  1 Bipolar (SR); 2 Substance dependence (SR); 1 Dyslexia (SR); 1 Dyspraxia (SR)  1 Eating Disorder (SR); 2 PTSD (SR) |
| Livingston et al. (2019 | **Mixed** (//)  **Survey** (Self-response) | 58 Formal diagnoses [AQ-10: 1-10]  19 Self-*identifying* [AQ-10: 3-10]  [89 of the total 136 participants (inc. those not relevant to this study) fell above the cut-off of 6] | *Possible IDD range varied [//] [ISCE: 0-7] (*”higher scores reflect higher educational attainment” – no further details).  Sample is described as highly educated. | 10 Co-developmental disorder (SR)  30 Anxiety (SR); 5 OCD (SR)  18 Depressive Disorder (SR)  1 Bipolar (SR); 1 Eating Disorder (SR)  1 PD (SR); 3 Trauma or Stress Disorder (SR) |
| Mantzalas et al. (2022) | **Qualitative** (//)  **Media Text Review** (Self-response) | 683 Self-reported [//] | *Not*  *provided* | *Not*  *provided* |
| Miller et al. (2021) | **Qualitative** (//)  **Survey** (Self-response) | 144 Self-reported [//] (formality was not checked and this is acknowledged) | *Not*  *provided* | 28 Depression (SR) (total); 25 Anxiety (SR) (total); 6 Dyslexia (SR) (total);; 5 ADHD (SR) (total) |
| Milner et al. (2019) | **Qualitative** (//)  **Single & Group Interviews** (Self-response) | 16 Formal diagnoses [//]  2 Self-*identifying;* 4 Parents (dyadic affiliations not stated) | *Not*  *provided* | *Not*  *provided* |
| Milton & Sims (2016) | **Qualitative** (//)  **Media Text Review** (Self-response) | 361 Self-reported [//] | *Not*  *provided* | *Not*  *provided* |
| Schneid & Raz (2020) | **Qualitative** (//)  **Single Interview** (Self-response) | 22 Formal diagnoses [//]  2 Self-*identifying* [//] | *Was not checked | *Not*  *provided* |
| Tint & Weiss (2018) | **Qualitative** (//)  **Group Interview** (Self-response) | 20 Formal diagnoses [//] | 20 Self-reported no IDD [//] | *Not*  *provided* |

## Section 2: Final Aggregate Report

Totals may not add to *100.00%* given rounding. Leeway of 0.01% was given.

Note that, in addition to the total number of participants used for the calculations below, there is also a “reported total value”. This means that the data is being compared to only the total number of participants for which a specific demographic factor was reported. Thus, studies that did “not provide” a specific piece of information (per the prior tables above) were not counted towards the participant tally for this value.

| **PARTICIPANT AGES** ***** | **Range: 15–79**  (729 participants are not provided a range) | **MEAN AGE:**  (1166 participants are not provided a mean)  [Right: Number of studies (N) provided] | Total sample (N = 28)  ‘Late-stage’ studies (N = 3)  ‘High-school’ studies (N = 2)  Total exclusive of ‘late-stage’ and ‘high-school’ studies (N = 17)  *Unknown* (N = 6) | **35.40 y**  **54.70 y**  **16.65 y**  **34.16 y**  **.** |
| --- | --- | --- | --- | --- |

| **GENDER** | Women (minimum)  **1059 (39.68%)** | Men (minimum)  **521 (19.52%)** | Other (minimum)  **103 (3.86%)** | *Unknown*  **982 (36.79%)** | Not applicable (Parent-input)  **4 (0.15%)** | | **Total**  **2669 (100%)** |
| --- | --- | --- | --- | --- | --- | --- | --- |
|  | *1059 (62.77%)* | *521 (30.88%)* | *103 (6.11%)* | ***.*** | *4 (0.24%)* | | Reported Total  *1687 (100%)* |
|  | ***.*** | *521 (34.19%)* | *103(6.76%)* | ***.*** | ***.*** | | *Mixed-gender studies 1524* |
| ***** Age data above applies only to age-relevant participants (/subsets). | | | | | |  | |

Please continue to the next page.

|  | | | | | **Total 2669 (100%)** | |
| --- | --- | --- | --- | --- | --- | --- |
|  |  |  |  | | *Reported Total *1146 (100%)* | |
| **NATIONAL RESIDENCE** | ****United Kingdom:** | Clearly demarcated (minimum)  *Clearly demarcated + high likelihood* (minimum) | **.**  ***.*** | | **881 (33.01%)**  **881 (76.88%)*  *1160 (43.46%)* | |
|  | **Oceania:** | Australia (minimum)  New Zealand (minimum) | **.**  **.** | | 115 (4.31%)  1 (0.04%) | |
|  |  |  |  | | **116 (4.35%)**  **116 (10.12%)* | |
|  | **North America:** | Canada (minimum)  United States (minimum)  *Unclear* (minimum) | **.**  **.**  **.** | | 21 (0.79%)  20 (0.75%)  28 (1.05%) | |
|  |  |  |  | | **69 (2.59%)**  **69 (6.02%)* | |
|  | **Europe:** | Western Europe  *Unclear* (minimum) | France (minimum)  *Unclear* (minimum)  **.** | | 31 (1.16%)  15 (0.56%)  7 (0.26%) | |
|  |  |  |  | | **53 (1.99%)**  **52 (4.62%)* | |
|  | **Asia:** | Southwest Asia | Israel (minimum) | | **25 (0.94%)**  **25 (2.18%)* | |
|  | **South America:** | *Unclear* (minimum) | **.** | | **1 (0.04%)**  **1 (0.09%)* | |
|  | **Africa:** | *Unclear* (minimum) | **.** | | **1 (0.04%)**  **1 (0.09%)* | |
|  | ***Unclear*** | ***.*** | **.** | | **1523 (57.06%)** | |
|  |  |  | | **Total 2669** | | **(100%)** |
|  |  |  | | *Reported Total *516* | | **(100%)* |
| **ETHNIC / CULTURAL IDENTITY** | **White** | White-British (minimum)  White-Australian (minimum)  *Unclear* (minimum) | | 110  81  286 | | 4.12%  3.03%  10.72% |
|  |  |  | | **477** | | **17.87%**  **92.44%* |
|  | **Mixed or Bi-racial** | *Unclear* (minimum) | | **29** | | **1.09%**  **5.62%* |
|  | **Asian** | *Unclear* (minimum) | | 8 | | **0.30%**  **1.55%* |
|  | **Black** | Black-British (minimum) | | **1** | | **0.04%**  **0.19%* |
|  | **Hispanic** | *Unclear* (minimum) | | **1** | | **0.04%**  **0.19%* |
|  | ***Unclear*** | ***.*** | | **2153** | | **80.67%** |

|  |  |  |  | **Total 2669 (100%)** |
| --- | --- | --- | --- | --- |
|  |  |  |  | *Reported Total *926 (100%)* |
| **EMPLOYMENT & RELATED**  (Cook et al., 2021 de facto listed as ‘unclear’ due to non-mutually exclusive categories) | **Occupation** | Active Student  Employed  *Unclear from above* (Minimum) | Secondary (minimum)  Tertiary (minimum)  Total  Full-time (minimum)  Part-time (minimum)  *********Unclear* (minimum)  Total  Full-time (minimum)  Part-time (minimum)  Total | 9 (0.34%)  166 (6.22%)  175 (6.56%)  **175 (18.90%)*  154 (5.77%)  86 (3.22%)  213 (7.98%)  453 (16.97%)  **453 (48.92%)*  11 (0.41%)  2 (0.07%)  13 (0.49%)  **13 (1.40%)* |
|  |  |  |  | **641 (24.02%)**  **641 (69.22%)* |
|  | **Unemployed or Unable to Work (minimum)** | **.** | **.** | **217 (8.13%)**  **217 (23.43%)* |
|  | **Retired (minimum)** | **.** | **.** | **19 (0.71%)**  **19 (2.05%)* |
|  | **Additional Categories** | *******Volunteers (minimum)  (Full-time ?) Carers (minimum) | **.**  **.** | **38 (1.42%)**  **38 (4.10%)*  **11 (0.41%)**  **11 (1.19%)* |
|  | ***Unclear*** | **.** | **.** | **1743 (65.31%)** |

** ‘Unclear’ employment covers vague, as well as self-employed or freelance, work categories.

| *** ‘Volunteers’ as its own category only references studies that delineated this from other forms of employment work. Thus, the ‘Employed’ category, in some cases, also encapsulates volunteers. | | |  | **Total 2669 (100%)** |
| --- | --- | --- | --- | --- |
|  |  |  |  | *Reported Total *793 (100%)* |
| **EDUCATION** | **Primary - Secondary** | Unqualified (Presumed < Secondary) (minimum) | **.** | **36 (1.35%)**  *36 (4.54%) |
|  | **Secondary Completed / Matric** | **(**Presumed) Mainstream (minimum)  (Reported) Special Needs (minimum) | 447 (16.75%)  11 (0.41%) | **458 (17.16%)**  *458 (57.76%) |
|  | **Tertiary (University or Trades Training)** | Undergraduate (minimum)  Postgraduate (minimum)  *Unspecified* (minimum) | 155 (5.81%)  112 (4.20%)  32 (1.20%) | **299 (11.20%)**  *299 (37.70%) |
|  | ***Unclear*** | **.** | **.** | **1876 (70.29%)** |

# Appendix 4: Complete List of Quote Extractions per Theme

The below final theme groups were derived from all A-Graded studies. Text provided in red reflects input from B-Graded studies, which were only inserted once final themes had already been derived. Articles have been numbered per Table 1 of the main article (i.e., as presented in Results).


**CAMOUFLAGING STRATEGIES**

**Theme 1. Masking**

**HULL ET AL 2017 (13)**

Masking encompasses […] a distinction between the respondent’s ‘true’ or ‘automatic’ behaviours, and what they present to the rest of the world.

**HICKEY ET AL 2018 (12)**

I was hiding my true self.

**LILLEY ET AL 2021 (16)**

At times, interviewees referred to an intentionally hidden self that they chose not to reveal in social encounters.

**Subtheme 1.1. Suppression**

Behavioural self-policing to eradicate or obfuscate existing behaviours or traits that indicate difference – the extreme of which reflects withdrawal, withholding, innocuity, and deference.

| **BARGIELA ET AL 2016 (1)**  Women commented that before having a diagnosis they would have ‘‘just kept quiet.” (F, AS)  **BERNADIN ET AL 2021 (2)**  “Instead of talking to them and maybe risking them being mean to me, maybe I just don’t say anything.” (F, AS, Minor)  **BOTHA ET AL 2020 (3)**  Concealment and withholding were directly talked about […]  **COOK ET AL 2021 (9)**  Some participants […] suppressing their more innate (and often autistic) verbal and non-verbal behaviours.  **HULL ET AL 2017 (13)**  Camouflaging was partly performed through suppressing, hiding, or otherwise controlling behaviours associated with ASC that were seen as inappropriate in the situation.  “I remain silent when I might otherwise have spoken, knowing that I can’t always tell whether or not my comments would be welcome.” (M, AS)  However, it is important to emphasise that not all respondents developed such structured rules for conversation; some simply had the goal of speaking as little as possible in order to get out of the interaction quickly: “[…] I avoid talking much […].” (F, AS)  **LEEDHAM ET AL 2020 (15)**  Sometimes avoidance of social contact was reported as being a preferable strategy.  **LIVINGSTON ET AL 2019 (17)**  Masking regulated existing behaviours, such as decreasing social behaviours thought by society to be undesirable (e.g., talking too much).  **MILLER ET AL 2021 (19)**  […] many participants emphasizing learning […] to suppress aspects of themselves. | **Innocuous Engagement Style** |
| --- | --- |
|  | **BARGIELA ET AL 2016 (1)**  Some women suggested that as their quiet and passive behaviours were seen as socially acceptable for girls, they had gone unnoticed, & proposed that had they been more disruptive they might have been noticed sooner.  They described how their perceived passivity, which they linked to their ASC, had led to unhealthy relationships and high-risk situations. One participant described feeling the need to ‘‘please, appease and apologise - do what you’re told’’ (F, AS)  **LEEDHAM ET AL 2020 (15)**  Previously stuck in a passive position […] [Participant] took control and ended the relationship with her husband. |
|  | **EXAMPLES** **OF SUPPRESSION** |
|  | - Keep quiet or speak as little as possible (perhaps an extension of innocuous engagement) - Suppress innate or automatic existing behaviours or aspects / traits of oneself - Avoidance of social contact - Suppress immediate reactions - Non-disclosure of personal information (inc. diagnosis) - Non-disclosure or -self-advocacy for own preferences - Minimising non-conventional interests and activities - Withhold distress and overwhelm (emotional, sensory- and fixation-based) - Minimising or suppressing of restricted repetitive, behaviours / OR / active self-monitoring and substitution of subtle stimming behaviours. |
| **SCHNEID & RAZ 2020 (21)**  […] “Or I reduce myself, like, to the point of suppressing my reactions.” (M, AS) | |

| **Facet 1.1.1: Hiding personal information, needs and preferences** |
| --- |
| **CROMPTON ET AL 2020 (10)**  Autistic people felt obligated to minimise or mask their natural behaviours and preferences in social situations with neurotypical people.  One of the most difficult things when your friends say ‘you should meet these people, they are great, let’s all go out to a pub’ […] on the other hand I don’t want to, I want everybody to go somewhere that is not noisy. But I also don’t want to be the person that makes us all go to a library . . . and speak in hushed tones. (F, AS)  **BOTHA ET AL 2020 (3)**  […] such as withholding diagnosis.  **COOK ET AL 2021 (9)**  Some participants selectively shared information about themselves [...] minimising more autistic or less conventional interests, characteristics and difficulties  **HULL ET AL 2017 (13)**  Minimising the amount of time they had to speak […] ensuring the ASC individual did not take over the conversation by talking about themselves or their own interests. […] Respondents were often aware that talking only about themselves and their interests was not socially acceptable and so developed strict rules to control their self-focused talk. For some, camouflaging also involved not divulging personal details about themselves.  “I say as little about myself as possible as the more I say, the more likely it is that I say something inappropriate.” (NB, AS)  “I do not talk about anything of interest to me […].” (F, AS)  **LILLEY ET AL 2021 (16)**  […] no longer feeling ashamed by their autistic traits or preferences.  **MILLER ET AL 2021 (19)**  ‘‘The worst part is not being able to stim when I need to’’ (F, AS)  **MILTON & SIMS 2016 (20)**  “I cannot talk about my real experience of life to most people, because they wouldn’t understand or be interested. That makes me feel, as the saying goes, ‘lonely in a room full of people’”. (M)  **DAVIDSON & HENDERSON 2010 (25)**  […] through a deliberate withholding of information […]  **FORSTER & PEARSON 2020 (26)**  “I certainly don’t talk about special interests.” (M, AS). |

| **Facet 1.1.2.: Hide distress** |
| --- |
| **BARGIELA ET AL 2016 (1)**  In contrast, to their good behaviour in school, these women recalled having had regular emotional ‘meltdowns’ at home after school: “I was unbearable with my mother, but at school I was perfect.” (F, AS).  **COLLIS ET AL 2022 (7)**  Participants also concealed distress in relation to their [*restricted, repetitive behaviours*] from others. This was particularly prominent with regard to the ‘get upset about minor changes to objects’ high order [*restricted, repetitive behaviours*]. [A participant] spoke of how they leant a book to a friend, only to watch “as if in slow motion” that friend break the spine of the book. Despite being in significant distress about this, they said: “It was like I didn’t say anything at the time.” […] because they knew that the other person would not understand the distress caused by their actions, they suppressed any outward expression of that distress.  **HARMENS ET AL 2022 (11)**  “When you have worked so hard to hold yourself together for so long, it is not easy to submit yourself to a [diagnostic] process.” (F)  **HULL ET AL 2017 (13)**  Camouflaging was frequently described as being mentally, physically, and emotionally draining; requiring […] management of discomfort.  Difficulties were often hidden behind the mask of camouflaging.  **LIVINGSTON ET AL 2019 (17)**  “We all have a hell of a lot of difficulties and just because we hide them doesn’t mean they don’t exist.” (F, AS)  **MILLER ET AL 2021 (19)**  Autistic participants specifically mentioned the interaction between cognitive and sensory processing, and masking. Some wrote about suppressing responses to sensory discomfort: ‘‘I frequently mask sensory differences such as pain due to sounds with too high pitch, too low pitch, or too high volume’’ (F)  **TINT AND WEISS 2018 (22)**  […] fluctuating service needs. That is, they described days when they are able to mask their difficulties […].  “So to the outside observer I’m sure I look passable - no one is going to expect that inside I’m driving myself crazy.”  **BALDWIN & COSTLEY 2015 (23)**  “Our pharmacy is in a busy shopping centre – the noise and activity is extreme. I have a meltdown (carefully hidden as much as possible) every week on average.”  “I experience cognitive shutdown and often meltdown as the day goes on, and have to just keep working.”  “I don’t feel like the daily struggles of adults with Asperger’s are well understood. I fight through (and hide, as best I can) a relentless struggle. I often feel like I just can’t keep going.” |

| **Facet 1.1.3. Suppression of stims**  **[With acknowledgement that two articles were dedicated specifically to stimming behaviours]** | |
| --- | --- |
| **COLLIS ET AL 2022 (7)**  […] resulting in a self-consciousness of their [*restricted, repetitive behaviours*], and leading, in some cases, to suppressing […] that behaviour. […] They were in some way […] reducing their [*restricted, repetitive behaviours*].  [A participant] spoke of how they would isolate their own repetitive behaviours by stopping the [*restricted, repetitive behaviours*] the moment even family members came by, and restarting the behaviour once they had left: “[…] sometimes when someone walks by […] I tend to stop and then let them carry on and then I can carry on sort of thing.”  Participants also discussed actively suppressing their [*restricted, repetitive behaviours*], using physical or mental restraint to prevent the [*restricted, repetitive behaviours*] from occurring. […] They talked of how they would reduce or completely suppress the behaviour […] eliminated the behaviour.  **HULL ET AL 2017 (13)**  Respondents described attempting to minimise their self-soothing or ‘stimming’ behaviours. […] “I prevent myself from doing any particularly visible or otherwise noticeable stims.” (F, AS)  Many respondents reported needing time to recover after camouflaging, where they could be alone and release all of the behaviours they had been suppressing.  **KAPP ET AL 2019 (14)**  Many […] attempted to suppress their stims. […] participants tried concealing stimming from view.  “‘If I thought anyone could see what I was doing, I could have stopped it.” (F, AS)  They hid […] stimming.  **MILLER ET AL 2021 (19)**  Several people wrote about suppressing their stims, for example: ‘‘many of us hide stimming […]’’ (F, AS) | **Role of Self-Monitoring** |
|  | **COLLIS ET AL 2022 (7)**  This self-policing of their behaviours […] for some participants constituted a pre-cursor to suppressing the [*restricted, repetitive behaviours*]. […]  […] substituting that behaviour. […] resulting in a constant self-monitoring of behaviours.  One strategy involved substituting their [*restricted, repetitive behaviours*] for a socially acceptable, or less obvious, alternative. […] so that they are more subtle or appropriate.  **COOK ET AL 2021 (9)**  “This [demonstrates hand flapping], works a lot better but it gets people’s attention a lot more so […] we do this [demonstrates hand-wringing], it’s a lot more socially acceptable”. (F, AS)  **HULL ET AL 2017 (13)**  These techniques included using objects as ‘props’ to meet sensory needs in a subtle way.  “I still find myself doing things like shaking my leg repeatedly without noticing, but don’t make any noises people would think are weird, don’t full-body shake (like with the leg but…all of me) […] (F, AS)  **KAPP ET AL 2019 (14)**  Other participants reported transmuting stims into a more socially acceptable form that provided similar feedback. […] e.g., stimming on her leg rather than on her desk at university.  Transmuted stimming  **MILLER ET AL 2021 (19)**  “Choosing discreet stims” (F, AS) |

**COOK ET AL 2021 (9)**

The camouflaging process also appeared to involve the dynamic monitoring of, and adaption to, cues in the social environment. Participants spoke of ‘constantly’ monitoring their own social behaviour to ensure they adequately performed camouflaging behaviours.

**HULL ET AL 2017 (13)**

Respondents described how these camouflaging techniques required intensive monitoring of the way they presented themselves.

**BRADLEY ET AL 2021 (4)**

Participants […] described needing to ‘‘constantly monitor’’ (M, AS) their behavior.

**Subtheme 1.2: Performance of Roles**

Part- or whole-performing of personas or contextual ‘roles’ that replace suppressed behaviours, and which fall to various degrees of incongruence with one’s sense of self. (e.g., performing characters, borrowing ‘personalities’, faking interests).

| **BARGIELA ET AL 2016 (1)**  […] costs of pretending to be someone else  ‘‘I honed something of a persona which was kind of bubbly and vivacious, and maybe a bit dim […]. So, I cultivated an image […] that was not ‘me’.’’ (F, AS)  Others reported having felt confused about their identity as a result of pretending to be someone else (F, AS)  Some had ‘‘acted neurotypical’ (F, AS)  Pretend to be interested in conversation topics.  **BERNADIN ET AL 2021 (2)**  [Participant (F, AS, Minor)] described negative consequences of this inauthenticity: “That is the downside of [camouflaging] […] you’re basically changing who you are […] they’re being friends with the person you’re changing to become.”  **BRADLEY ET AL 2021 (4)**  They describe […*a*] ‘social mode’’ (M, AS), ‘‘people suit’’ (M, AS) […] that they would use to cope with everyday social situations as if playing a ‘‘game’’ (F, S-AS), ‘‘part’’ (F, S-AS), or ‘‘role’’ (F, AS).  **CAGE ET AL 2018 (5)**  “I can fake neurotypical behaviour pretty well.”  **HARMENS ET AL 2022 (11)**  “I became so many different people that I felt I’d lost any sense of my own identity.” (F)  **HICKEY ET AL 2018 (12)**  Interaction retained a performance quality.  **HULL ET AL 2017 (13)**  Masking enabled respondents to present a different identity to the outside world […] concealing one’s actual personality. […] In some cases, this went as far as portraying an entirely different character, and several respondents likened it to acting or performing a role, complete with costumes.  “I camouflage by putting on a character … I treat my clothes rather like costumes, and certain items of clothing help me to uphold certain personality characteristics of which character I am on that occasion […] They are all me at the core, but they are edited versions of me.” (F, AS)  Lying about who they were.  “I feel like the weight of a black cloud is hanging on me having to be this fake version of me” (F, AS)  “Literally never being able to be myself.” (F, AS)  “It becomes very isolating because even when I’m with other people I feel like I’ve just been playing a part.” (F, AS)  “Just pretend to be interested in what people are saying.” (F, AS)  **LEEDHAM ET AL 2020 (15)**  Some participants took on a persona that was ego dystonic in order to fit in:  “[I wore] different clothes to everything that I wore at home … I hated this person that I put on.” (F, AS)  Disguising their true selves.  **LILLEY ET AL 2021 (16)**  Interviewees variously described themselves as ‘playing a role’ (M, AS), ‘playing with identities’ (F, AS) or ‘trying on different personalities’ (F, AS). This description of role-playing was most frequent in early adulthood: […] [Participant (M, AS)] said that at university, “I was mister footloose and fancy free […].” | **Situational Roles** |
| --- | --- |
|  | **BARGIELA ET AL 2016 (1)**  Several recalled being regarded as the ‘‘teacher’s pet’’ (F, AS) or the ‘model pupil’.  **HULL ET AL 2017 (13)**  “I have a repertoire of roles for: cafe work, bar work, uni, various groups of friends, etc.” (F, AS)  Respondents often felt they were playing so many different roles, it was hard to keep track of their authentic sense of identity.  **The character or aspects of the role could change across different situations:**  **LILLEY ET AL 2021 (16)**  [Participant (F, AS)] described being ‘on the outer’ at high school, explaining “I’d flit around different groups”.  ‘I was kind of playing the student role’ (M, AS).  **SCHNEID & RAZ 2020 (21)**  “I have to be the alpha male. Like, taking the role of the actor, or the instructor, or the Emcee, or the mediator” (M, AS)  **DAVIDSON & HENDERSON 2010 (25)**  “I have **preprogrammed**/learned behaviors for church, meals, restaurants, casual, semi-casual, formal situations. With these programs as a cover-up, I am able to accomplish much that is considered normal, successful, desirable. But this is a shell, and within it I’m bombarded and puzzled.”  [Participant] has managed to perfect her protective skills as a performer to the extent that: ‘There are times when I am in professional mode and the person I am with would probably never believe I was autistic […]”  **FORSTER & PEARSON 2020 (26)**  I had to practice how to be a student in mainstream education, it is like playing a role.” (M. AS). |
|  | **Feeling like a fragmented patchwork** |
|  | **BRADLEY ET AL 2021 (4)**  The effort it took to ‘‘pretend’’ […] they are ‘‘neurotypical’’.  Many described having lost the ‘‘real me’’ […] or feeling ‘‘fake’’ […] and having ‘‘little of ‘me’ left’’ (F, AS) because they were ‘‘a patchwork of acts’’ (M, AS).  **LILLEY ET AL 2021 (16)**  “And what was my life before diagnosis? It was bits and pieces of disconnected things […] And there was no centre.” (F, AS) |
|  | **EXAMPLES OF PERFORMATIVITY** |
|  | - Pretend to be a different (/neurotypical) person (changing facet’s of one’s identity or personality) - Develop a context-specific persona(s), identity variation or character(s) that can be ‘performed’ as a ‘role’ - Pretend to be interested in a topic - Fake interests - Use outfits or items of clothing to uphold a persona - Take on a blasé or nonchalant air - Make use of humour to distract or detract from suspicions - Adopt a pre-given role such as the “good student” - Use socially accepted ailments as excuses. |
| Sometimes this role-playing was accompanied by marked identity fluidity […] “My mother said afterwards, before you left home, it was like you didn’t have a personality” (M, AS)  “I’d just kind of been all these characters and I didn’t know who I was” (F, AS)  “I didn’t know about masking or camouflaging but I knew I could not present my true self to the world” (F, AS)  “I’m like some performer . . . like a one trick pony that is trying to fake my way through life.” (M, AS)  **LIVINGSTON ET AL 2019 (17)**  Compensation often involved some deception (e.g., faking interests).  Participants reported that compensation—widely described as “putting on a performance”—resulted in a diminished and uncertain sense of self.  **MILTON & SIMS 2016 (20)**  “I have developed an ‘act’ to be ’normal’, which has allowed me to interact with people” (M) […] Some talked of a learnt performance of normalcy.  Others described how creating a facade of normality that subsumed their sense of authentic self.  **SCHNEID & RAZ 2020 (21)**  Taking the role of the other.  Many respondents described in this context how social functioning will always be artificial for them, a kind of going through the motions […] Many respondents thus felt that their social behavior was not really theirs […] but something contrived.  Respondents thus viewed much of their social behaviour [… as] A special personality that needs to be built in order to face the world while keeping the “real me” intact.  **BALDWIN & COSTLEY 2015 (23)**  “I have learned to act […] I still think what I like, but mask it behind a smile.”  “I had to study acting to appear as normal for the customers.”  **DACHEZ & NDOBO 2018 (24)**  The participants also often use theatre vocabulary, explaining that they have the impression that they are playing a role, wearing a costume, being on the stage, like [Participant (F, AS)]: “I realised that the school playground was like a theatre stage, I had to act in a play”.  However, this is a strategy that can be exhausting in the long term. In fact, this **borrowed** personality requires constant effort, and prevents the person from being in tune with him/herself.  “[…] knowing how to play a “character” in brackets could help quite a bit.” (M, AS)  “I often react with humour. Humour is one of my ways of reacting or explaining things in general. For example, if someone says to me “Oh you can speak?” I answer “Oh yes, you see how well I can pretend, eh!”” (F, AS)  **DAVIDSON & HENDERSON 2010 (25)**  The ‘qualified deception’ repertoire: Providing rich insights on the complexities of passing, the qualified deception repertoire “is based on the idea that activities involving varying degrees of fabrication, either via a reconstruction of the details […]  [Participant] also writes of putting on a front and ‘acting out’ social ritual […]  In certain times and places, however, it becomes impossible to blend in, and drawing attention to aspects of one’s difference might be essential to the successful, if not fully disclosed, negotiation of social geographies. Referring to an everyday encounter with a clerk in a grocery store, [Participant], for example, explains that “she may have to repeat things to me because of my problem of deciphering sounds in a noisy environment. I say, “sorry, I have trouble hearing in a noisy area”—I don’t tell her about my diagnosis of autism.’ Other less contested or stigmatized conditions can also be used as a front, and [participant] describes referring to migraines as “an excuse that is accepted by almost everyone.”  “If I pretend to be rubbing my temples, as if I am warding off a headache, people do not react oddly when they see me.”  When people put pressure on her to eat: “I claim to be allergic. And if they still keep pestering me with questions about my eating habits.” | |

**LINK TO IMITATION:**

**HULL ET AL 2017 (13)**

One way to easily identify the appropriate role to play was to mimic the behaviours of others during a social interaction.

**Theme 2: ‘Tools’ for a Growing Repertoire**

The means by which new and more complex social skills are learned or developed.

**FORSTER & PEARSON 2020 (26)**

These statements were indicative of participants learning to socialise like one may learn to ride a bike. Instead of relying on ‘implicit’ social signals that neurotypicals pick up on more easily, here the participants treat socialising as a skillset to be learned.

[…] had worked hard to develop their understanding of others.

**Subtheme 2.1: Imitation**

| **BARGIELA ET AL 2016 (1)**  Strategies that women learnt from […] other people.  Social mimicry was another strategy used in social situations. However, young women reported that mimicry was automatic and unconscious. […] “I honestly didn’t know I was doing it [social mimicry] until I was diagnosed, but when I read about it, it made perfect sense. I copy speech patterns and certain body language.” (F, AS)  Some young women had noticed that they would quickly pick up accents from other people […] “I automatically mimic what other people are doing, what people are saying, how people say things, I went on [Girl Guide] camps…and I would come back with strong accents. But I can’t consciously put on an accent…my way of coping is that I mimic.” (F, AS)  Many had found that the effort required to process consciously people’s behaviours and later act them out, was exhausting.  First, the role of social mimicry was considered: “There’s potential for you copying a guy’s flirtatious behaviour without realising that’s what you’re doing.” (F, AS).  **BRADLEY ET AL 2021 (4)**  Participants described […] how they spend time ‘‘watching others’’ (F, AS), ‘‘mimicking social behaviours’’ (F, AS).  **COOK ET AL 2021 (9)**  Other participants described carefully observing people (autistic and non-autistic) engaging in social interactions from afar, carefully noting the manner in which they engaged with and responded to each other. Some reported focusing in particular on the behaviours of socially valued individuals.  “Everyone seemed to really like her and they always used to say things like, ‘Oh, she’s so happy, she’s so funny’, […] So I changed my laugh […] to make it a bit more like hers.” (F, AS)  **CROMPTON ET AL 2020 (10)**  [Non-autistic people] “never had to study autistic people in the same way I study them.” (F, AS)  **HICKEY ET AL 2018 (12)**  After conceptualising their difference, participants engaged in a process of studying their peers and imitating their social performance  “You emulate things […] you actually outwardly become exactly like everybody else.” (M, AS)  “And I tried to smile like they smiled.” (F, AS)  **HULL ET AL 2017 (13)**  Behaviours could be copied directly from the person in front of them, or could be identified and learned from observing others interacting […] Some respondents went as far as to copy clothing style, mannerisms, and even interests from others.  “I try to copy socially successful people by trying to imitate their speech and body language and trying to understand their interests.” (M, AS) | **The iterative process** |
| --- | --- |
|  | **COOK ET AL 2021 (9)**  Participants’ idiosyncratic repertoires of camouflaging behaviours were developed and refined through an iterative process.  Other participants described […] trialling these behaviours.  “ I started practising my laugh.” (F, AS)  **LEEDHAM ET AL 2020 (15)**  [Other strategies] were practised with the aim of learning social rules and appearing ‘normal’ when making comparative evaluations to people.  **LIVINGSTON ET AL 2019 (17)**  A range of internal factors were found to drive compensation and modulate strategy success. Compensation was linked to individual differences in autism-related (e.g., detail focus) and non-autism-related (e.g., intelligence) processes, to plan, execute, and refine strategies.  These individuals now found social situations easier […] through refining strategies.  **FORSTER & PEARSON 2020 (26)**  “I feel like I’ve taught myself just through loads of very strict, conscious practice over the years. |
|  | **EXAMPLES OF IMITATION** |
|  | - Copy other’s speech patterns (inc. accents) and body language during an interaction - Do what other people do (mannerisms) and say what other people *phrase*, dress as others dress - Copy the interests others express - After the interaction, act out, re-enact or outright trial / practice the behaviour of others - Observe and study (socially valued individuals (?)) as a third party, and copy aspects (e.g., manner of laughing, manner of smiling) - Track all the minute details of social interactions - Copy phrases and behaviours from different media sources, including from fictional characters. |
| **LEEDHAM ET AL 2020 (15)**  Mimicking served a survival function.  **MILLER ET AL 2021 (19)**  […] mirroring behavior, expressions, dress sense, and speech: “I copy people’s phrases, and dress sense too.” (F)  **SCHNEID & RAZ 2020 (21)**  “I looked outside all the time to see how other kids are behaving.” (F, AS) […]  “In a normal social situation with other normal people … mimic the others.” (F, AS)  “To overcome and understand what others think […] In order to imitate behaviors. To imitate behaviors … The autistic perception works in such a way that they see, they have understanding in lots and lots of details, […] These marginal details. Focusing on the marginal." (M, AS)  **TINT & WEISS 2018 (22)**  Engage in social observation and mimicry.  **DACHEZ & NDOBO 2018 (24)**  […] by imitating their peers or concealing their difficulties.  “I realised that mimicking […] could help quite a bit.” (M, AS) | |
| **Facet 2.1.1. Learning from Media** | |
| **BARGIELA ET AL 2016 (1)**  Strategies that women learnt from various media […] Many women described actively learning how to ‘mask’ from different media sources including characters on television, magazines, […] and novels: ‘‘They’d have the right behaviour for certain things, so ‘If you want this, you should do this’.’’ (F, AS).  Another woman learnt phrases and facial expressions from fictional literature in order to manage more unpleasant situations, such as bullying. ‘‘When I was being bullied, there’s this book by Ellen Montgomery and the character Emily, whenever somebody is horrible to her…she just looks at them, and because of her expression they go away.’’ (F, AS)  **HULL ET AL 2017 (13)**  Behaviours […] could be identified and learned […] from watching television and films.  **LILLEY ET AL 2021 (16)**  I spent so much of my life living with this paradigm that I’d made out of books that this is somehow what life is meant to be about.  He learned many of his behaviours from ‘Hollywood films’ and ‘reading different books’.  **LIVINGSTON ET AL 2019 (17)**  “I repeat myself or use tv/film phrases and sometimes say things which are out of place.” (F, AS) | |

**Subtheme 2.2: Active Social Training**

| **BARGIELA ET AL 2016 (1)**  learning how to ‘mask’ from […] books on body language […]  Other women used skills they had actively learnt, such as ‘‘a guide to being assertive’’ (F, AS) provided by a counsellor and skills learnt in their jobs.  **MILTON & SIMS 2016 (20)**  [Specific social interactions]: “The autistic spectrum is so wide that I know many of you will be reading this thinking, ‘Oh, I don’t need social skills training’, but some people do, especially around bullying.” (F)  **SCHNEID & RAZ 2020 (21)**  “There was nobody there, if I look back, that told me, listen, let's help you learn how to communicate. Let's help you navigate, understand what is correct, in various social issues, how to manage situations.” (F, S-AS) […] The respondent stressed that while she wanted someone to instruct her, at the same time such instructing felt coercive. This was an inevitable source of conflict for her: […] “Sometime I get insulted … like, what is wrong with me?” (F, AS)  **BALDWIN & COSTLEY 2015 (23)**  77% indicated that they had not received enough support to make friends and develop social skills.  I would like to have some basic knowledge of social skill etiquette so I can be with a group of friends comfortably, without feeling self-conscious about making a mistake.  I would like to learn skills for overcoming shyness.  Reflective of these statements, 62% of participants reported that they needed support to improve their social skills, and 44% expressed a need for support to help deal with bullying and discrimination.  ... spoke of their desire for greater understanding by others […] and help with developing skills and strategies in areas such as communication and coping with change.  A number of the open comments recorded across the survey suggested that the women had actively sought (or, at least, identified a need) to ‘learn’ aspects of communication and social behaviour that would enable them to relate to others in a way that would be perceived as appropriate. |
| --- |

**Theme 3: Cognitive Strategies**

| **BRADLEY ET AL 2021 (4)**  [Participants] describe adopting a set of “rules”. (F, S-AS)  **HULL ET AL 2017 (13)**  By camouflaging and using structured techniques, respondents could reduce some of this uncertainty.  The other aspects of camouflaging centre around developing explicit strategies […] Respondents often described these techniques as ‘rules’ […].  Different expressions were identified as important for different situations, and so many respondents described keeping a mental list of how to behave depending where they were.  [Adjusted behaviours] included non-verbal and verbal signs of interest in the interaction, which were also used to encourage others to continue speaking and so take the pressure off the ASC individual to respond appropriately.  “I’m not good at knowing when it’s my turn and I also tend to just blurt out things or keep talking when I should have stopped, so I prep myself always in social situations to have a reminder or tag or internal buzzer about not speaking too much and trying to do more listening, nodding, agreeing.” (F, AS)  Respondents reported developing rules or guidelines […] These were more generalised and so could be prepared ahead of time and applied to different situations. These camouflaging strategies were used to help the ASC individual get through ‘small talk’ or more in-depth conversations […]  [*Explanations*] included minimising the amount of time they had to speak, giving them more time to prepare things to say.  “I make generic comments rather than offering specific ones that might reveal my more unusual traits.” (M, AS)  **LIVINGSTON ET AL 2019 (17)**  Compensation involved intellectual and executive functions to regulate social behaviour, such as intellectually conceived patterns about social norms […] and switching between social rules.  Differing levels of compensatory strategy […] Many strategies, involving shallow compensation were simple and inflexible (e.g., laughing after joke cues).  **DAVIDSON & HENDERSON 2010 (25)**  “Every social encounter requires constant decoding and then selection of an appropriate response […]” | **‘Ask questions’ rule** |
| --- | --- |
|  | **HULL ET AL 2017 (13)**  One rule was to ask questions of the other people.  “I’ve recently tried to institute a rule about asking more “you” questions - how did that make you feel, what did you do next, what do you think about a given thing - instead of “me” or “I” statements.” (M, AS)  “I try to ask them about the things they like, question after question, to keep conversation going but sometimes it doesn’t work and they leave me.” (F, AS)  “I find asking questions is the best deflection.” (F, AS)  **COOK ET AL 2021 (9)**  “I did my usual party trick of she asks me a question and I just flip it back and I give her answers and flip it back and say, “And you?” It’s my way of keeping the conversation going”. (NB, AS)  **LIVINGSTON ET AL 2019 (17)**  (e.g., asking others questions about themselves) |
|  | **EXAMPLES OF COGNITIVE STRATEGIES** |
|  | - Adopt a set of cognitively derived, explicit rules and guidelines to structure engagement in social situations - Allocate specific facial expressions or behaviours to specific contexts /OR/ different rules for different contexts - Use signs of interest (nodding, agreeing) to prompt the other person to keep speaking - Use direct questions (and ‘you’ statements) to prompt the other person to keep speaking - Set an internal ‘reminder’ or ‘timer’ for when to stop speaking - minimising the amount of speaking time - Make general rather than specific statements - Respond to demarcated prompts (such as joke cues) - Pre-plan topics of conversation and prepare responses ahead of time (scripting) - Use mental equations and patterns to interpret a situation or predict what is up-coming - Create sub-patterns of behaviour for interaction with each person - Make use of digital communication to allow for response planning |
| **Subtheme 3.1: Scripting** | |
| **BARGIELA ET AL 2016**  The use of an online platform had made communication easier for some young women. For example, in normal face-to-face communication, one would be expected to ‘read’ body language, tone of voice and facial expression, so ‘‘If all we have is typing for each other, then it’s completely equal’’ (F, AS). Women talked about being able to express themselves more clearly when they didn’t have the pressure and anxiety to respond immediately, as with a face-to-face conversation. Further, use of messaging was also an easier and less awkward medium to express difficult emotions and access support from their friends.  **COOK ET AL 2021 (9)**  [Participant (NB, AS)] described being aware that they had difficulty maintaining conversations and used a scripted phrase to overcome this […]  **HULL ET AL 2017 (13)**  “I tend to think of one or two questions to ask the person and most people are so happy just to talk about themselves […].” (F, AS)  Respondents also described spending time before an interaction to prepare topics of conversation, including questions to ask, anecdotes to relate, and potential responses to others. These […] reassured them that they would have structured ‘scripts.  “I usually also think up stories and how whole conversations might go before I have them so I have responses practiced as well as potential things to say.” (F, AS)  **LILLEY ET AL 2021 (16)**  [Participant (M, AS)] commented that when he was ‘dating’ he was ‘probably scripting everything’.  **LIVINGSTON ET AL 2019 (17)**  Preplanning social niceties  **MILTON & SIMS 2016 (20)**  Others talked of how they needed to prepare themselves for tackling an unforgiving environment.  **BALDWIN & COSTLEY 2015 (23)**  “I […] developed a list of public responses I refer to. […]”  **HOWARD & SEDGEWICK 2021 (27)**  In contrast to the strong dislike for phone calls, Written Communication was consistently a preferred form of communication […] Thinking time […] was the most given reason for preferring written communication – writing gave people ‘time to think about what I want to say’, provided the opportunity to ‘arrange my thoughts and ask all my questions before sending’ and the ability to ‘make sure it looks good and reads well’ so their words ‘have the effect that [they] are looking for’. […]  Structured communication. Another major factor in favour of written communication was it being a ‘structured way to interact’ compared to face-to-face or phone calls, with some participants saying that they “research templates for what [they] need” or “find phrases [they] can borrow”. | |
| **Subtheme 3.2: Modulation** | |
| **BARGIELA ET AL 2016 (1)**  [Participants] shared their regret and anger at having ‘‘tried to be good’’ (F, AS) for so long.  **BERNADIN ET AL 2021 (2)**  “So, I’ll um, I’ll try and act more extroverted […] Just so they can see that maybe I’m not the, I don’t know, the angry quiet girl that never talks.” (F, AS, Minor)  **BRADLEY ET AL 2021 (4)**  Participants described their experiences of camouflaging as the need to “adapt behaviour’’ […]” (M, AS)  These ‘‘rules’’ were needed to help participants get ‘‘all of the social cues right’’ (F, AS) ‘‘have the right facial expression’’ (F, AS) and respond in the ‘‘right way’’ (F, AS).  **CAGE & TROXELL-WHITMAN 2019 (6)**  Some participants used camouflaging as a tool to manage others’ impressions by presenting a particular image of the self, for example to demonstrate their competence and skills.  **COOK ET Al 2018 (8)**  Many [parents[ reported how well their daughters coped or how they made adjustments to their behaviour in order to fit in.  **COOK ET AL 2021 (9)**  Some participants sought to promote their social image via positive attributes, reporting attempts to be perceived as ‘similar’ to their social partner, ‘friendly’, ‘nice’ and ‘intelligent’ […] engaging in social behaviours that demonstrated their positive attributes and highlighted similarities between themselves and their social partner. They avoided behaviours that potentially signalled undesirable traits […].  “What I’m trying to do is to smooth my tone of voice out . . . and make it sound less choppy which seems closer to what most neurotypical people do.” (NB, AS).  Some participants selectively shared information about themselves, emphasising their more normative interests, and characteristics, or circumstances and minimising more autistic or less conventional interests.  “I do make eye contact with people.” (M, AS)  “So, I had to train myself to do eye contact.” (F, AS).  **HULL ET AL 2017 (13)**  Masking […] covered up those parts of themselves they were not happy with.  Explicit, compensatory strategies were reported by many respondents as a vital way to improve non-verbal communication with others.  Forcing and maintaining appropriate eye contact, or attempting to look as close to another’s eyes as possible, was a common compensatory technique reported. Respondents also made an effort to display facial expressions of emotion or interest, even if they didn’t feel this inside. | **The Role of Monitoring** |
|  | **COOK ET AL 2021 (9)**  At the same time, they described closely examining their social partner’s interpersonal cues for signs of, for example, engagement and interest or boredom and discomfort. They then adjusted their behaviours in response to these cues:  “[The social partner] is nodding and appears to be engaged which is why I carried on with conversation. If she started to look bored and not terribly interested, I would have gone to a different topic of conversation, probably her.” (M, AS)  Other participants described cognitive aspects of camouflaging such as monitoring their performance and the social cues of others as being challenging and energy consuming.  **HULL ET AL 2017 (13)**  The combination of controlled behaviour and appropriate conversation produced through camouflaging was often described as essential during social interactions […]  Camouflaging therefore often involved a constant monitoring of the situation, as if training oneself in self-monitoring, self-awareness, and monitoring others’ reactions.  **TINT & WEISS 2018 (22)**  “Yeah, I’m totally aware when someone sees me as weird.” “Exactly! It’s like your spidey sense is tingling. It’s like, uh-oh, that person has received weirdness now I have to amend my presentation so that I can pass better.”  **FORSTER & PEARSON 2020 (26)**  “Thankfully I can catch myself a lot better now.” (M, AS)  During the interviews participants expressed engagement with frequent self-monitoring during social interactions, suggesting that they found it difficult to ‘be themselves’: “I’ll often get worried are they bored of what I’m saying and are they tired of me blathering on. |
|  | **EXAMPLES OF MODULATION** |
|  | - Adjust behaviours to a specific conception of what the public would consider the ‘good’ way or ‘right’ way - Play up behaviours that present one’s competence, intelligence or skills - Promote positive attributes that others would see as ‘similar’, friendly, or nice - Smoothen out tone of voice - Selectively share normative interests and characteristics - Make eye contact with social partner, or look in the vicinity of the eyes - Display facial expressions (e.g., smiling) and emotions of interest - Over-emphasise responses so as to not appear as having a ‘flat affect’ |
| “I look in people’s eyes when I first meet them[…] because I know you’re supposed to.” (F, AS)  “I try to look people in the eye and make faces that fit the situation.” (NB, AS)  Many respondents noted that their preferred levels of emotional expression and body language did not match those of others around them, and so over-emphasised these behaviours in order to communicate better.  “My autistic lack of non-verbal signals are read as hostility, arrogance or indifference by people, so I have to act the good will that I genuinely feel.” (F, AS)  **LIVINGSTON ET AL 2019 (17)**  Making eye contact  Whereas compensation generated new behaviours, masking regulated existing behaviours, such as […] increasing behaviours thought to be desirable (e.g., smiling).  **SCHNEID & RAZ 2020 (21)**  Impression management was a much broader concept which in addition to camouflaging also included techniques for inclusion assuming a social performance that builds on and accentuates one's inner repertory of traits and beliefs.  **DACHEZ & NDOBO 2018 (24)**  “I try to erase the autistic aspects […] to always look people in the eyes too, to pay attention.” (M, AS)  [Participant (M, AS)] has adapted his way of speaking: “I’ve completely reformatted my language, I make mistakes and I swear”.  “Especially for facial expressions, I can force myself for a whole evening, but it will really hurt when I get home, I’ll have sore muscles.” (F, AS)  **HOWARD & SEDGEWICK 2021 (27)**  […] such as “masking tone of voice”, having to do an “emotional performance with my face.”  **MILNER ET AL. 2019 (28)**  “You’ve probably noticed she makes eye contact but it’s, it’s a bit clunky you know, but she’s learnt to do that.” (Parent of F, AS) | |
| **Subtheme 3.3: Mental Equations** | |
| **BARGIELA ET AL 2016 (1)**  ‘‘It’s very draining trying to figure out everything all the time, everything is more like on a manual, you’ve got to use one of those computers where you have to type every command in.’’ (F, AS)  **BRADLEY ET AL 2021 (4)**  Participants explained there being ‘‘so many rules of what to do in each situation’’ (F, AS), that their brain was described as ‘‘constantly working and ticking’’ (F, AS), ‘‘building patterns and sub-patterns to describe every situation based on every person” (M, AS) […]: ‘‘It is EXHAUSTING. It’s like trying to solve mathematical equations in your head all day long while carrying on as normal’’ (F, AS). | **Bespoke Patterns** |
|  | **BRADLEY ET AL 2021 (4)**  ‘‘building patterns and sub-patterns […] based on every person” (M, AS)  **LIVINGSTON ET AL 2019 (17)**  “It’s almost a case of systematically storing little patterns in each person and the context, so I can refer to it in future.” (F, AS)  **MILLER ET AL 2021 (19)**  In addition, one participant talked about their use of memory as a social tool for masking, ‘‘I remember lots of details about people so I have things that I know they want to talk about’’ |
| **HULL ET AL 2017 (13)**  Camouflaging was frequently described as […] draining; requiring intensive concentration.  “I will be incredibly anxious. It’s like studying for an exam, constantly on edge trying to predict what others will say and do.” (F, AS)  **LEEDHAM ET AL 2020 (15)**  “[Other people] […] just seemed to do things without the whole thought process that I have to go through.” (F, AS)  **LIVINGSTON ET AL 2019 (17)**  Deep compensation, involving complex and flexible strategies, contributed to some improvements in social cognition. Some participants reported using pattern detection and internal data modelling (gesture + facial expression + context = particular mental state) to understand others.  “I think I could make ‘all the right choices’ in social situations if I could choose offline with more time to reflect and from afar, but real situations are far trickier.” (M, S-AS)  “think I observe patterns in behaviour and then try to transfer this. So if a person is behaving x/y/z types of ways, they could be feeling or thinking what so and so people had felt.” (F, AS)  **DACHEZ & NDOBO 2018 (24)**  [Participant (M, AS) explains having spent a great deal of time studying facial expressions and having become a real “mentalist”: “You know, it’s always in intellectual mode, it’s not intuitive, but then it becomes impressive because you’ve got 20 years of practice. You’ve got the memory, that’s what’s amazing. You stack it up … It’s like semantic computing tools. As the database expands, the system becomes more refined and you become more efficient”.  **FORSTER & PEARSON 2020 (26)**  […] ‘learning’ about how to ‘read’ other people and fit in […] Some of the participants discussed how they had learned over time how to interact with others: “The more you get to know someone the easier it is to read them … It’s like a formula … It’s something you learn.” (F, AS).  **HOWARD & SEDGEWICK 2021 (27)**  […[ “being on the phone feels starkly alone with a disembodied voice” (showing difficulties interpreting voice without other cues).  Some participants linked these challenges directly to aspects of being autistic – especially difficulty inferring social contexts, such as how it is ‘extremely difficult to interpret’ someone’s tone or intentions ‘without other signs to point you in the right direction’. Other participants said that ‘it’s better when I can see the person I’m communicating with’ because then you ‘see the facial and body language of the speaker’. | |

**Theme 4: Use of Others**

| **BRADLEY ET AL 2021 (4)**  Participants described […] attaching themselves to someone more social ‘‘to give the illusion of being sociable’’ (F, S-AS)  **CROMPTON ET AL 2020 (10)**  Neurotypical people could be beneficial in a social situation. […] they mentioned the benefits of neurotypical people being able to explain to the autistic person in a 1:1 context what was happening in a group conversation, or wider social event: “I can be like “what is going on here?” and then tell them about something, and they can tell me “this is what is happening”’.” (F, AS)  **LEEDHAM ET AL 2020 (15)**  “… he’ll [husband] now take the lead in situations where he knows I’m not comfortable.” (F, AS)  **LIVINGSTON ET AL 2019 (17)**  Participants described examples of accommodation during childhood (e.g., parent communicating on their behalf), which had enabled them to compensate sufficiently.  **SCHNEID & RAZ 2020 (21)**  “I have colleagues at work who are friends and if there's a situation that I do not understand, I pick up the phone and say: please explain to me what in fact this was about. The nuances. I feel I didn't understand it. If it's a really good friend she would explain it to me. It's the difference of asking for help in a focused and fully aware manner, versus being lost without even knowing why you're lost.” (F, AS)  **DAVIDSON & HENDERSON 2010 (25)**  […] enlisting the help of an unwitting other when she wants to visit a gallery or a town she could never tackle on her own: “I’ve been able to pretend that I want their company while in fact what I’ve needed is an escort.”  **HOWARD & SEDGEWICK 2021 (27)**  **Supported Communication:** […] The other side of the difficulties many people reported were the support they often called on to enable them to engage in communication. Both informal and formal supports were mentioned, […] Many participants said that they ‘get someone else to check’ high stakes written communications, such as emails or letters dealing with healthcare or financial matters, and that often in face-to-face situations they ‘want someone with [them] for moral support’. |
| --- |

**Theme 5: ‘Unhealthy’ Practices**

| **BARGIELA ET AL 2016 (1)**  Another woman described using alcohol as a way to ‘‘free me up to maintain my neurotypical mask’’ (F, AS) in situations where she needed to pretend to be interested in conversation topics, such as television programmes that she did not like  **BRADLEY ET AL 2021 (4)**  However, others described using alcohol to get ‘‘through social events’’, as it helped them ‘‘feel less anxious and self-aware’’ (F, AS). | **Faking the behaviour** |
| --- | --- |
|  | **CROMPTON ET AL 2020 (10)**  “I’m the only autistic one and I find it very difficult to keep up with conversations and I lose words . . . the others think I’m drunk sometimes (although I’ve not been drinking), and I let them think that.” (F, AS) |
| **MILLER ET AL 2021 (19)**  Autistic participants also specifically mentioned ‘‘dangerous’’ coping mechanisms. […] Another wrote about substance use, ‘‘I used to use alcohol to do it, but recently I gave it up so masking has become more difficult’’ (F).  Autistic participants also specifically mentioned ‘‘dangerous’’ coping mechanisms. One wrote about restricted eating, ‘‘In order to try and fit in somewhere. I have suffered with Anorexia Nervosa for 8 years’’ (F). […] These comments support the idea that there may be something specific to the experience of masking in autistic people that might make it particularly harmful. | |

**Theme 6: Selective Settings**

| **BARGIELA ET AL 2016 (1)**  In contrast, a number of young women said they felt more at ease in their friendships with males. This was not thought to be related to biological sex, but to society ‘allowing’ men to be more straightforward, and this being a communication style that suited women with ASC better: ‘‘I just feel so much more comfortable with men because they’re more, you can take them at face value and its not that fear of them judging you or having alternative motives and thoughts and they kind of say things straight.’’ (F, 19-22, Artist, UK)  **COOK ET AL 2018 (8)**  Where successful friendships had been formed, they were often with other girls with special needs, or who were different in some way.[…] this tendency was also found in girls in mainstream settings who tended to gravitate towards other girls with autism without realising it.  “Well they were kind of just free and didn’t really care too much what other people thought, which is a lot like what I am. So they kind of just resemble me, in a different way, so that’s probably why I got along with them so much.” (F, AS, Minor)  The tendency to befriend others with autism or other differences enabled them to feel accepted and to be themselves.  **HICKEY ET AL 2018 (12)**  Prior to diagnosis […] engage with people, usually in quite structured settings. They sometimes joined clubs related to interests […]  “It was a structured thing and I felt rather more comfortable with that.” (M, AS)  The focus on something tangible (e.g., a book) facilitated the use of social skills in the absence of an overt focus on social skills: “That’s where we’re focused on – it might be book club and we’re focused on the book. But if you listen to the discussion on the book, it’s very flowing and we can be really supportive.” (M, AS)  **LILLEY ET AL 2021 (16)**  The idea of self-reinvention was highlighted by interviewees. [Participant (F, AS)] described moving overseas as “another opportunity to reinvent myself.”  […] commented on frequent changes of employment, explaining, “I seemed to run into difficulties […] rather than try and resolve them, I’d leave and find another job.”  **LIVINGSTON ET AL 2019 (17)**  “I have spent most of my adult life in the UK. Rules and social norms are different here…a much easier fit for many people on the spectrum, because some of the things that come more naturally to us are valued in British culture—a certain amount of reserve, reticence, not treating everyone you meet like they are instantly a friend.” (F, AS)  […] pursuing environments that supported strategy success  They reported seeking environments that were accommodating of their autistic characteristics, such as workplaces in which non-social abilities were more important than social skills.  **MILTON & SIMS 2016 (20)**  On occasion, however, these intrinsic needs were set within a social context with comments referring to society as being too fast paced or not accommodating a slow and deliberate style of processing or thinking, with some commenting on a preference for a rural lifestyle.  Many talked of finding safe and accepting social spaces within their lives in order to retreat from the social spaces where they felt ostracised.  **DACHEZ & NDOBO 2018 (24)**  Twenty-one participants mentioned having searched for support from friends who are “atypical”, either because of their age, their socio-professional class, their nationality or their way of functioning (autistic or exceptionally gifted friends, for example).   - They have a way of communicating that resembles that of people with autism, which makes talking easier. - Their experience resembles that of the person with autism, which enables them to find common ground and to validate their own experience through someone else’s. - These atypical people are also excluded and stigmatised, which creates a natural reciprocal empathy. As [Respondent (F, AS)] explains: “In primary school, I was friends with all those who had been rejected by the others, all the losers, all the odd ones, the strange, the crazy ones. (...) We were automatically excluded, so we trusted each other”. - They are so different that there can be no social comparison, which prevents the person with autism from being in a situation where they risk feeling out of place or even devalued. [Respondent (M, AS)] explains: “And all my friends without Asperger’s, it’s very funny, they’re either much older people or children, who I get along with very well. When the generations are relatively similar, you can easily compare yourself to others and it’s very miserable.”   **FORSTER & PEARSON 2020 (26)**  Several of the participants spoke about finding people who are ‘like-minded’(2.3), as they are easier to spend time with: ‘Knowing where to be and where I’ll find people similar, or meeting friends of friends who would be like minded.’ (M, AS). |
| --- |

**CONTEXTUAL MODERATORS OF CAMOUFLAGING**

**Theme 1: Unclear and Unexpected Cues**

| **LEEDHAM ET AL 2020 (15)**  Efforts to ‘fit in’ were influenced by several factors, including confusing social interaction attempts and perceived failures.  **LIVINGSTON ET AL 2019 (17)**  Compensatory strategies had an upper limit because they did not function in all situations or were too slow and inflexible in fast-moving social interaction. […] responding to unexpected turns of conversation, could not be achieved via the secondary route of compensation.  [*Shallow compensation*] […] transferred poorly to new contexts.  “I am stuck when I meet people who have no interests and extreme extroverts.” (F, AS)  [*Compensation*] […] unstructured social settings (e.g., parties), were demanding.  **SCHNEID & RAZ 2020 (21)**  “… Some of these times were really ambivalent – I didn't want to talk with anybody because I didn't understand.” (F, S-AS)  **BALDWIN & COSTLEY 2015 (23)**  I don’t like interacting with people I can’t ‘read’. |
| --- |

**Theme 2: Number of People**

| **BARGIELA ET AL 2016 (1)**  Socialising as part of large groups was reported as challenging by all the young women interviewed.  **COLLIS ET AL 2022 (7)**  […] isolating their [restricted, repetitive behaviours] to specific environments, typically ones in which they were alone, out of sight of others.  [Participant] would isolate their own repetitive behaviours by stopping the [restricted, repetitive behaviours] the moment even family members came by, and restarting the behaviour once they had left.  “I do get a little bit shy or even embarrassed when someone else is present.” (AS)  **HICKEY ET AL 2018 (12)**  [I] find it difficult to talk to people especially in groups and things  **LIVINGSTON ET AL 2019 (17)**  Additionally, group situations, involving multiple social cues […] were more demanding on compensatory resources than one-to-one structured interaction (e.g., doctor’s appointment). Therefore, many individuals reported “passing” as neurotypical in environments with low demands, but appearing socially atypical in those with higher demands.  **SCHNEID & RAZ 2020 (21)**  “Social grouping brings out my autism.” (F, AS) |
| --- |

**Theme 3: Distress and Overwhelm**

| **BRADLEY ET AL 2021 (4)**  Participants needed time to ‘‘recover’’ (F, AS) and ‘‘recharge’’ (M, AS). If participants were unable to take recovery days, they described not bring able to ‘‘cope’’, putting them at risk of a ‘‘meltdown’’.  **HULL ET AL 2017 (13)**  “Sometimes, when I have had to do a lot of camouflaging in a high stress environment, I feel as though I’ve lost track of who I really am, and that my actual self is floating somewhere above me like a balloon.” (F, AS)  **LIVINGSTON ET AL 2019 (17)**  Compensation was therefore more difficult when distracted or stressed. […] [*shallow compensation strategies*] were also so less effective when stressed […]  “If I am having a bad day, all strategies go out the window, socialisation is no longer a priority.” (F, AS)  “Sensory environment. It can wipe out 100% of my ‘coping energy’ in moments.” (F, AS)  Environmental demands, such as social interactions in loud and bright rooms, made it difficult for participants to use compensatory strategies.  For most participants, when demands (e.g., living independently) increased in adulthood, their compensatory strategies became insufficient or their autistic characteristics impaired their daily functioning. In some cases, life-changing events (e.g., death of a partner) led to such compensation breakdown.  **MILLER ET AL 2021 (19)**  “I can manage to exist in an NT world as long as I’m ok mentally, if I’m stressed.it all turns to jelly, as do I.” (M)  Others wrote about sensory processing difficulties making masking harder: ‘‘Sensory processing difficulties, particularly auditory, make masking tougher’’ (NB)  **TINT & WEISS 2018 (22)**  […] fluctuating service needs. That is, they described days when they are able to mask their difficulties […] as compared to other days when they require more support, mostly due to anxiety and other mental health concerns.  Some participants also described reaching a point of exhaustion when they “break down” (e.g. experience fits of yelling and crying) and are unable to mask their difficulties any longer  **DAVIDSON & HENDERSON 2010 (25)**  […] authors recognize that in the course of day-to-day life, there will be occasions when one’s social camouflage breaks down for one reason or another, causing a temporary failure in the ability to pass. In stressful circumstances, the person with autism might find comfort by arm-flapping, verbalizing or rocking, or other behaviours considered sufficiently ‘abnormal’ in public that they will attract more than second glances from passers by.  **FORSTER & PEARSON 2020 (26)**  “Sometimes I don’t have the energy to really try (to interpret meanings).” (M, AS). Here the participant notes the psychologically demanding nature of social interactions. |
| --- |

**Theme 4: Duration of Camouflaging**

| **BOTHA ET AL 2020 (3)**  “[…] act every single waking moment of our lives.” (NB, AS)  **BRADLEY ET AL 2021 [*when in public*] (4)**  “For autistics, we have to camouflage all the time, even with people we know pretty well.” (F, S-AS)  Participants reporting to ‘‘always’’ camouflage.  […] their brain was described as ‘‘constantly working” (F, AS)  “When the interactions are short and meaningless.” (M, AS).  **CAGE & TROXELL-WHITMAN (2019) (6)**  To get through situations […] as quickly as possible”  **COOK ET AL 2021 (9)**  […] ‘constantly’ monitoring their own social behaviour.  **HARMENS ET AL 2022 (11)**  I could appear fairly ‘‘normal’’ for stretches of time.  **HULL ET AL 2017 (13)**  The extent to which this happened could vary […] although some respondents described camouflaging at all times.  The longer a camouflaging session continued, the harder it became to maintain the intended level of camouflaging.  **LILLEY ET AL 2021 (16)**  constantly performing to meet societal expectations  **LIVINGSTON ET AL 2019 (17)**  Compensation was therefore […] difficult to sustain  “The inability to mask or compensate beyond the initial stages of a relationship.” (M, AS)  **MANTZALAS ET AL 2022 (18)**  “We need to remind ourselves to unmask.”  **MILLER ET AL 2021 (19)**  One autistic participant gave an account of how pervasive and all- encompassing masking can be, ‘‘Life is masking, masking is life’’ (F)  **MILTON & SIMS 2016 (20)**  “[…] shed the masks that have to be worn every day.”  Some talked of the minor advantages to passing within specific, but temporary, social interactions.  **TINT & WEISS 2018 (22)**  “I can turn on my normal long enough to sit through a half hour appointment.”  **DACHEZ & NDOBO 2018 (24)**  “It’s a mask, […] Especially for facial expressions, I can force myself for a whole evening, but it will really hurt. |
| --- |

#

# Appendix 5: GRADE-CERQual Framework

**Confidence in the Evidence from Reviews of Qualitative Research** (<https://www.cerqual.org/>)

The study findings were assessed per the following four domains of consideration:

**(1) Methodological Limitations**: Findings are challenged when the original studies have problems in the way they were designed or conducted.

A full quality-grid scoring process was undertaken. For any studies in which there was significant concern (especially regarding the analytic methods or ethics of a study), they were automatically designated as a ‘B-graded’ study and did not contribute to the initial drafting of core findings/themes (and thus core findings exist independent of such studies). While such a semi-exclusionary grading system helped manage significant methodological concerns – it must be noted that the significant limitation to the overall data cohort is in demographic reporting (as reported in the first section of Results). There was a large inconsistency and lack of reporting on details such as nationality, employment, race, and socio-economic status. However, the strong bias of data towards White, female and UK-based participants does provide some form of a counter-balancing homogeneity to the sample, and allows some degree of context to be asserted around the findings. This stands even if the transferability of the results is questioned. Furthermore, it is unclear how many studies prompted participants to speak about camouflaging and masking, versus waiting for participants to organically brings up such a topic on their own.

**(2) Relevance:** The body of evidence from the primary studies needs to be directly applicable (versus substantively different) to the context of the current review question(s).

All studies met the bare minimum inclusion criteria in order to ensure relevance to the parameters of the review and its questions. However, it is fair to state that some studies only reached ‘partial relevance’ given their (a) focus on women only, rather than all AS adults, or (b) where ‘adult’ samples extended down to age 15, when the inclusion cut-off was age 16. While not all studies were centred specifically on camouflaging, they all provided input on strategies AS adults use to fit in socially and the motivations therearound.

**(3) Coherence:** There needs to be a clear and cogent fit between the different sets of data (without the presence of significant contradictions or ambiguity).

This is one of the largest strengths of the review – in that there was remarkable agreement across the data in the core findings. No significant contradictions were found in the data. Any significant inconsistencies were themselves embedded in findings (such as how different perspectives could be explained by whether the participant was discussing a time before diagnosis, just after diagnosis, or years after diagnosis following integration with the AS community). While the transferability of the data set may be questioned, the contents of the existing literature base appear congruent.

**(4) Adequacy of the Data**: A rich quantity of data needs to have contributed to the results (versus only coming from a small number of studies or participants).

The 28 studies, consisting of a total of 2450 participants, is considered to a fully adequate amount of data to have synthesised, as it is neither a small cohort (considering review work) nor an insignificant amount of contributing individuals. This is especially considering it is only fairly recently that the topic of camouflaging has been proliferating.

We overall hold **MODERATE CERTAINTY** in the results of the study (given that, while we hold high certainty in the relevance, coherence and adequacy of the data, the methodological difficulties surrounding demographic reporting make it difficult to make decisive claims around the transferability of the findings).

While this decision is ultimately a subjective consideration or judgement for rating all facets of the review study, the GRADE-CERQual framework provides a structured and transparent means of reporting and motivating why such a decision has been made. While such reporting would usually be conducted per theme or output category – much of the limiting considerations for the data was in the demographic reporting, and as such was relatively consistent across all themes found.
